# Comprehensive analysis indicated that NDE1 is a potential biomarker for pan‐cancer and promotes bladder cancer progression

**DOI:** 10.1002/cam4.6931

**Published:** 2024-03-11

**Authors:** Peihan Wang, Jinzhuo Ning, Wu Chen, Fan Zou, Weimin Yu, Ting Rao, Fan Cheng

**Affiliations:** ^1^ Department of Urology, Hubei International Scientific and Technological Cooperation Base of Immunotherapy Renmin Hospital of Wuhan University Wuhan P.R. China

**Keywords:** bladder cancer, BLCA, immune, NDE1, pan‐cancer

## Abstract

**Background:**

The nuclear distribution E homologue 1 (NDE1) is a crucial dynein binding partner. The NDE1 protein has the potential to disrupt the normal functioning of centrosomes, leading to a compromised ability to generate spindles and ensure precise separation of chromosomes during cell division. The potential consequences of this phenomenon include genomic instability, malignant transformation and the proliferation of neoplastic growths. However, studies examining the connection between NDE1 and cancer is still very rare.

**Methods:**

The expression level, prognostic impact, gene change, DNA methylation, protein interaction, mRNA m6A modification, ceRNA network, associated gene and function enrichment, and immune‐related effects of NDE1 in pan‐cancer were examined using a range of online analytic tools and the R software package. The CCK‐8 test, transwell assay, scratch assay and colony formation assay were used to confirm the effects of NDE1 on the proliferation, invasion and metastasis of bladder cancer cells.

**Results:**

Numerous tumour types have elevated NDE1, which is linked to a bad prognosis. NDE1 is an excellent diagnostic tool for many different types of cancer. Numerous malignancies have been linked to genetic changes in NDE1. NDE1 was connected to TMB, MSI, several immunological checkpoint genes and immune cell infiltration. NDE1 is linked to a number of immunological subtypes. NDE1 could affect how well immunotherapy works to treat different types of cancer. NDE1 was mostly associated with cell cycle, chromosomal segregation, DNA replication and mitotic segregation, according to GO and KEGG analyses. NDE1 physically binds to PAFAH1B1 and DCTN1, respectively. The proliferation, invasion and metastasis of bladder cancer cells may be prevented by NDE1 knockdown. Furthermore, knockdown of NDE1 promoted the apoptosis of bladder cancer cells.

**Conclusion:**

High expression of NDE1 is present in a variety of tumours, which is linked to a bad prognosis for cancer. Knockdown of NDE1 inhibited the proliferation, invasion and metastasis of bladder cancer cells, and promoted the apoptosis. For a number of malignancies, NDE1 may be a biomarker for immunotherapy and prognosis.

## 1 INTRODUCTION

Cytoplasmic dynein‐1 (dynein) is involved in a diverse array of functions, such as intracellular transportation of cargo, construction of the spindle apparatus and modulation of several physiological and pathological processes. These processes include cell proliferation, cell cycle regulation, signal transduction, inflammatory response and immunological response.^1^, ^2^ The predominant emphasis of previous studies investigating the relationship between abnormal regulation of dynein activity and pathological situations has mostly centred on neurodegenerative illnesses, such as Parkinson's syndrome.^3^ Recent research has shown that dynein plays a significant role in regulating cellular proliferation and invasion, and its involvement has been associated with the development and prognosis of many tumours.^4^ For instance, some studies have discovered that dynein may influence the formation of mitotic spindles, which may contribute to the development and malignant phenotype of colorectal cancer and cervical cancer.^5^ According to Bartl et al., stabilising dynein accelerated the growth of various tumours.^6^ Dynein cytoplasmic heavy chain 1 has also been shown to be strongly linked to hepatocellular carcinoma's poor prognosis and immune infiltration.^7^

Dynein's full activity replies on its regulatory partners. For instance, in the past 10 years, it has been shown that Lis1, dynactin and a class of proteins known as activation adapters are involved in the activation and control of dynein.^8^ The internal retrograde movement of membrane organelles along the microtubule network is regulated by dynein, together with its regulatory proteins NDE1/NDEL1 and Lis1.^9^ Dynein's major binding partner, Nuclear Distribution E Homologue 1 (NDE1), is involved in a number of physiological and pathological processes, including the formation and evolution of the gyral cortex, brain inflammation and neurodegenerative diseases.^10^, ^11^, ^12^

Centrosome abnormalities may cause genomic instability by altering chromosomal segregation during mitosis, which is directly associated with the development of cancer.^13^, ^14^ According to one theory, NDE1 participates in the assembly of a group of centrosome localisation proteins, and incorrect expression of these proteins causes aberrant cell division. Additionally, the complex has the ability to interact with the cancer‐related protein p78/MCRS1, which raises the risk of genetic malignant transformation.^15^ Hence, it was postulated that NDE1 could be implicated in the pathogenesis and advancement of tumours.

Studies examining the connection between NDE1 and cancer are still very rare or even nonexistent. We investigated NDE1's expression level, prognostic impact, gene alteration, DNA methylation, protein interaction, m6A mRNA modification, ceRNA network, related gene and function enrichment, and immune‐related effects in pan‐cancer. Furthermore, we corroborated a subset of the findings from the bioinformatics analysis by experimental methods such as co‐immunoprecipitation, reverse transcription polymerase chain reaction (RT‐PCR) and other relevant techniques. In order to fill the research gap pertaining to the impact of NDE1 on the genesis and progression of cancer, our study also included experimental investigations aimed at elucidating the role of NDE1 in facilitating the proliferation, migration and invasion of bladder cancer cells. New targets for the prevention and treatment of numerous malignancies, including bladder cancer, might be found as a result of our investigation.

## 2 METHODS AND MATERIALS

### 2.1 Analysis of NDE1 expression level

NDE1 expression levels in various human body tissues are examined using the GTEx, HPA and FANTOM5 three transcriptome research databases on the human protein mapping (THPA) online website (https://www.proteinatlas.org/). In several human tissues, the normalised expression (NX) of NDE1 was estimated. The cancer genome atlas (TCGA) and genotype‐organisation express (GTEx) tumour gene expression data were downloaded from the UCSC Xena database (https://xenabrowser.net/datapages/). R software (version 3.6.4) was used to visualise the data, and a schematic diagram of NDE1 expression in cancer and paired adjacent tissues was created. The online sangerbox website (http://www.sangerbox.com/) was used to create violin plots showing NDE1 expression levels in various malignancies. The violin diagram was created after using the GEPIA2 portal to analyse the relationship between NDE1 expression and the clinical stage of the tumour.

### 2.2 Prognostic analysis

The TCGA database's cancer survival data were examined using the GEPIA2 portal, and the correlations between NDE1 and overall survival in various malignancies as well as between NDE1 and disease‐free survival were determined. Additionally, we established a Cox proportional hazards regression mode using the coxph function of the R package ‘survival’ (version 3.2–7) and did statistical tests for prognostic significance using the Logrank test.

### 2.3 Receiver operator characteristic (ROC) curve illustration

The ‘pROC’ package of the R programme (v1.17.0.1) was used to compute and analyse the data, and the ‘ggplot2’ package was used to generate the ROC curve. The expression data for NDE1 were obtained from the TCGA database.

### 2.4 Analysing genetic changes

We examined the location, kind and degree of DNA methylation of NDE1 gene changes using the cbioportal platform (https://www.cbioportal.org/) and the UALCAN web portal (http://ualcan.path.uab.edu/index.html/).

### 2.5 Analysis of m6A modification and protein interaction

NDE1 protein interaction with other proteins was analysed using the STRING portal (https://www.string‐db.org/), and the NDE1 m6A modification site in mRNA was predicted using the SRAMP prediction website (http://www.cuilab.cn/sramp). Co‐immunoprecipitation assay was used to verify the direct interaction between proteins.

### 2.6 Examination of immune‐related characteristics

From the TCGA database, we took the NDE1 expression data, processed it using log2(X + 1) and then took the gene expression profile of each tumour included in the data. Using the Timer and Quantizeq methods of the R package IOBR (version 0.99.9), respectively, immune cell infiltration in NDE1 was assessed. We used the corr.test function of the R package ‘psych’ (version 2.1.6) to compute the immune infiltration score. The plot(x, y) function was used to construct scatter plots in order to more clearly illustrate the link between NDE1 gene expression level and immune cell infiltration. The Immune Checkpoint Genes module of Sangerbox Online was then used to examine the link between NDE1 and several immune checkpoint genes. Tumour purity statistics for several tumours were previously summarised by Thorsson et al.^16^ The connection between tumour purity and NDE1 expression levels was then determined by integrating these data with NDE1 expression levels in the relevant samples. The relationship between NDE1 expression levels and homologous recombination deficit (HRD) was also examined. The impact of NDE1 on sensitivity to immunotherapy was examined using the TISMO database. Additionally, the expression of NDE1 was examined to see whether it linked with MSI or TMB using the R package ‘fmsb’. Additionally, we examined the connections between NDE1 and tumour immune subtypes and molecular subtypes using the TISIDB portal's ‘subtypes’ module. In addition, several immunological pathways and activities were linked to NDE1 by KEGG and GO enrichment studies.

### 2.7 Analysis of the NDE1‐related genes

To locate and display the genes strongly associated with NDE1, we obtained data from the GEPIA2 database and the TIMER2 database. To investigate the biological roles of NDE1‐related genes, investigations of the gene ontology (GO) and the Kyoto Encyclopaedia of Genes and Genomes (KEGG) were carried out.

### 2.8 Construction of competing endogenous RNA(ceRNA) networks

To look for NDE1 upstream miRNAs, information was collected from the miRDB, miRWalk, miRabel and TrgetScan databases. The lncRNA/circRNA‐miRNA‐NDE1 regulatory network was built using the two miRNAs with the highest TargetScan scores.

### 2.9 Culture of cells

Procell Life Science and Technology sold the T24 and 5637 cell lines for human bladder urothelial cancer. In 5A media, the T24 cell line was grown. In 1640 medium, the 5637 cell line was grown. 10% FBS (fetal bovine serum) and standard concentration of penicillin/streptomycin antibiotics were added to the medium.

### 2.10 Small interfering RNA (siRNA) transfection

The siRNA was ordered from Shanghai's OBiO Technology. In order to silence NDE1, we created two siRNA sequences. Following is a list of the siRNA sequences: si‐NDE1#1, 5′‐CGAUCAUGUCUCUCGAAGACU‐3′ sense and 5′‐UCUUCGAGAGACAUGAUCGUG‐3′ antisense; si‐NDE1#2, 5′‐GCAGCUGCAACAAAUUGAAAC‐3′ sense and 5′‐GCAGCUGCAACAAAUUGAAAC‐3′ antisense. According to the manufacturer's recommendations, cells were split into one negative control group and two knockdown groups before being transiently transfected with Lipofectamine 3000 in six‐well plates with T24 and 5637 cells. The effectiveness of NDE1 knockdown was evaluated using a western blot test.

### 2.11 MicroRNA (miRNA) transfection

The miRNA mimics and negative control mimics (NC mimics) was ordered from Wuhan Heyuan Biotechnology Co. LTD. Transfection was performed using Lipofectamine 2000 according to the manufacturer's protocol. Cells were treated by 48 h starvation for further analyses.

### 2.12 Quantitative real‐time PCR

Total RNA was extracted from T24 cells using TRIzol Reagent (Invitrogen Life Technologies, Carlsbad, CA, USA), and the RNA purity was determined using a DU800 UV/Vis Spectrophotometer (Beckman Coulter, CA, USA). Subsequently, total cellular RNAs were reversed transcribed into cDNA using a reverse transcription reagent kit (Toyobo, Osaka, Japan). Real‐time quantitative PCR was performed via an Applied Biosystems SYBR Green Mix Kit and an ABI 7900 Real‐Time PCR System (Applied Biosystems Life Technologies, Foster City, CA, USA). The expression of NDE1 mRNA was normalised to GAPDH, respectively. The relative amount of mRNA was calculated using the 2‐∆∆Ct method. The primer sequences used are shown in Table 1.

**TABLE 1:** RT‐PCR primer sequences.

Gene	Primer sequences (5′‐3′)
NDE1	F: TCAGTCCCCTCCCGATA
	R: AGCTCATTCTCCGCTTCA
GAPDH	F: ACAGCAACAGGGTGGTGGAC
	R: TTTGAGGGTGCAGCGAACTT

### 2.13 CCK‐8 assay

Cell growth was compared using the CCK‐8 assay. 96‐well plates with 100 L of medium per well were seeded with about 5103 cells per well. Each well received 10 g of the CCK‐8 solution, which was then added and incubated in the dark for 2 h before the absorbance was measured at 450 nm.

### 2.14 Cell scratch assay

In six‐well plates, T24 and 5637 cells were expanded until they completely occupied the field of view (100 percent confluence). We scratched the cells in a straight line using a sterile 200‐μL pipette tip. The scratch width was then measured under a microscope after 0 and 12 h, and the results were then compared.

### 2.15 Transwell assay

Using a polycarbonate membrane, we split the 24‐well plate into two upper and lower sections. The top layer received media without serum, while the bottom layer received media containing 10% serum. In the top layer, cells were sown and cultured for 24 h. The capacity of cell invasion and migration was then examined by counting the number of cells in the bottom layer after the cells had been fixed with paraformaldehyde and stained with crystal violet solution.

### 2.16 Assay for colony development

After 7 days of culture, T24 and 5637 cells were transferred with the medium onto six‐well plates at a density of 500 cells per well, fixed with 4% paraformaldehyde for 20 min, stained with 4% crystal violet dye and then counted by colonies.

### 2.17 Western blot analysis

A combination of protease, phosphatase inhibitor and RIPA lysis solution was used to lyse T24 and 5637 cells. Cellular proteins were isolated and then added to SDS‐PAGE gels for electrophoresis before being transferred to PVDF membranes. According to the target protein's molecular weight, the primary antibody was added and incubated overnight, followed by the addition of the secondary antibody and a 2‐h wait at room temperature before the photographs were taken.

### 2.18 Co‐immunoprecipitation (co‐IP)

T24 cells were first lysed using mild cell lysate under nondenaturing conditions, followed by addition of agarose beads conjugated to the relevant antibody to the cell lysate and incubation. The precipitated proteins were eluted using the eluate, and these proteins were identified and analysed by western blotting.

### 2.19 Flow cytometry analysis

For cell apoptosis analysis, cells were double‐stained with Annexin V‐fluorescein isothiocyanate and PI and tested using flow cytometry.

### 2.20 Analytical statistics

The R language and databases were used to automatically analyse non‐experimental data, whereas GraphPad Prism 9 was used to analyse experimental data.

## 3 RESULTS

### 3.1 Analysis of NDE1 expression levels in cancers

**FIGURE 1:**
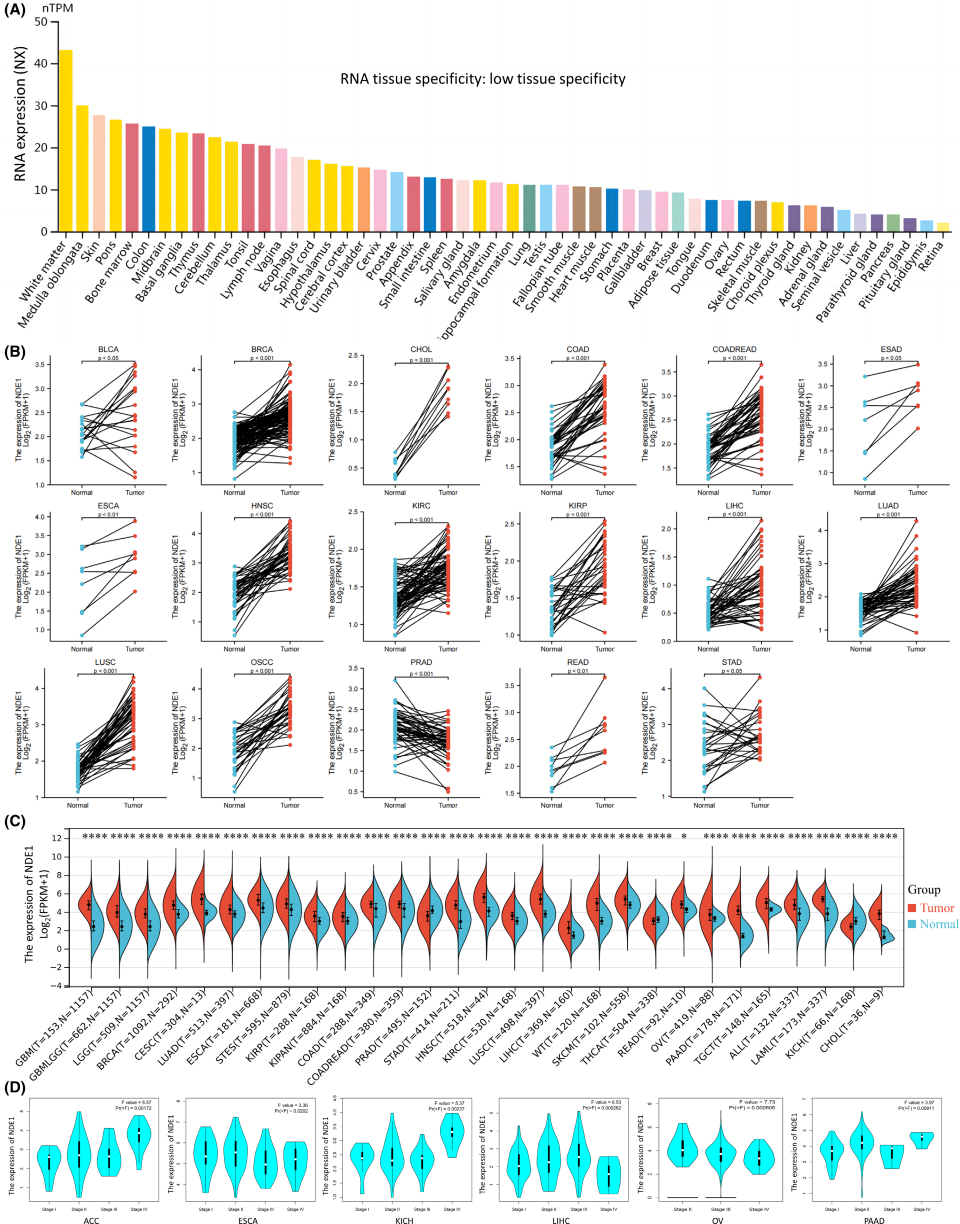
NDE1 expression levels in normal tissues and cancers. (A) Consensus normalised expression (NX) levels of NDE1 in dozens of normal tissue types and blood cell types generated from three transcriptome datasets (GTEx, HPA and FANTOM5); (B) Pan‐cancer differential expression of NDE1 in paired tumour and adjacent normal tissues in indicated tumour types from TCGA database; (C) NDE1 mRNA expression levels in different tumours and corresponding normal tissues obtained from TCGA and GTEx databases; (D) The correlation between NDE1 expression and tumour stages in various tumours in TCGA datasets. *p < 0.05; **p < 0.01; ***p < 0.001 and ****p < 0.0001.

It was discovered that the expression level of NDE1 mRNA in healthy human subjects was low (NX < 50) after using the Human Protein Atlas to examine the normalised expression level of NDE1 in different tumour tissues and paracancerous tissues. NDE1 RNA has little tissue specificity, despite the fact that NDE1 expression can be seen in the majority of tissues (NX > 10 in most tissues) (Figure 1A). NDE1 was, however, shown to be significantly expressed in the majority of malignancies after examination of matched samples (cancer and paracancerous tissues) from the TCGA and GEO databases. NDE1 was specifically shown to be substantially expressed in the following cancer species in paired samples: BLCA, BRCA, CHOL, COAD, COADREAD, ESAD, ESCA, HNSC, KIRC, KIRP, LIHC, LUAD, LUSC, OSCC, READ and STAD, but was only weakly expressed in PAAD (Figure 1B). Further analysis of NDE1 mRNA expression in TCGA and GEO databases showed that NDE1 was highly expressed in 26 types of cancers (ALL, BRCA, CESC, CHOL, COAD, COADREAD, ESCA, GBM, GBMLGG, HNSC, KIPAN, KIRC, KIRP, LAML, LGG, LIHC, LUAD, LUSC, OV, PAAD, READ, SKCM, STES, STAD, TGCT and WT) and lowly expressed in three types of cancers (KICH, PRAD and THCA). The GEPIA2 portal examined the association between NDE1 expression and tumour grade. While NDE1 expression was inversely connected with tumour grade in ESCA and OV, it was favourably correlated with tumour stage in ACC, KICH, LIHC and PAAD (Figure 1D). In summary, it can be seen that NDE1 has a significantly increased level of expression in a majority of tumours, and its association with the clinical stage of many malignancies has been established by us.

### 3.2 Evaluation of NDE1's prognostic relevance

**FIGURE 2:**
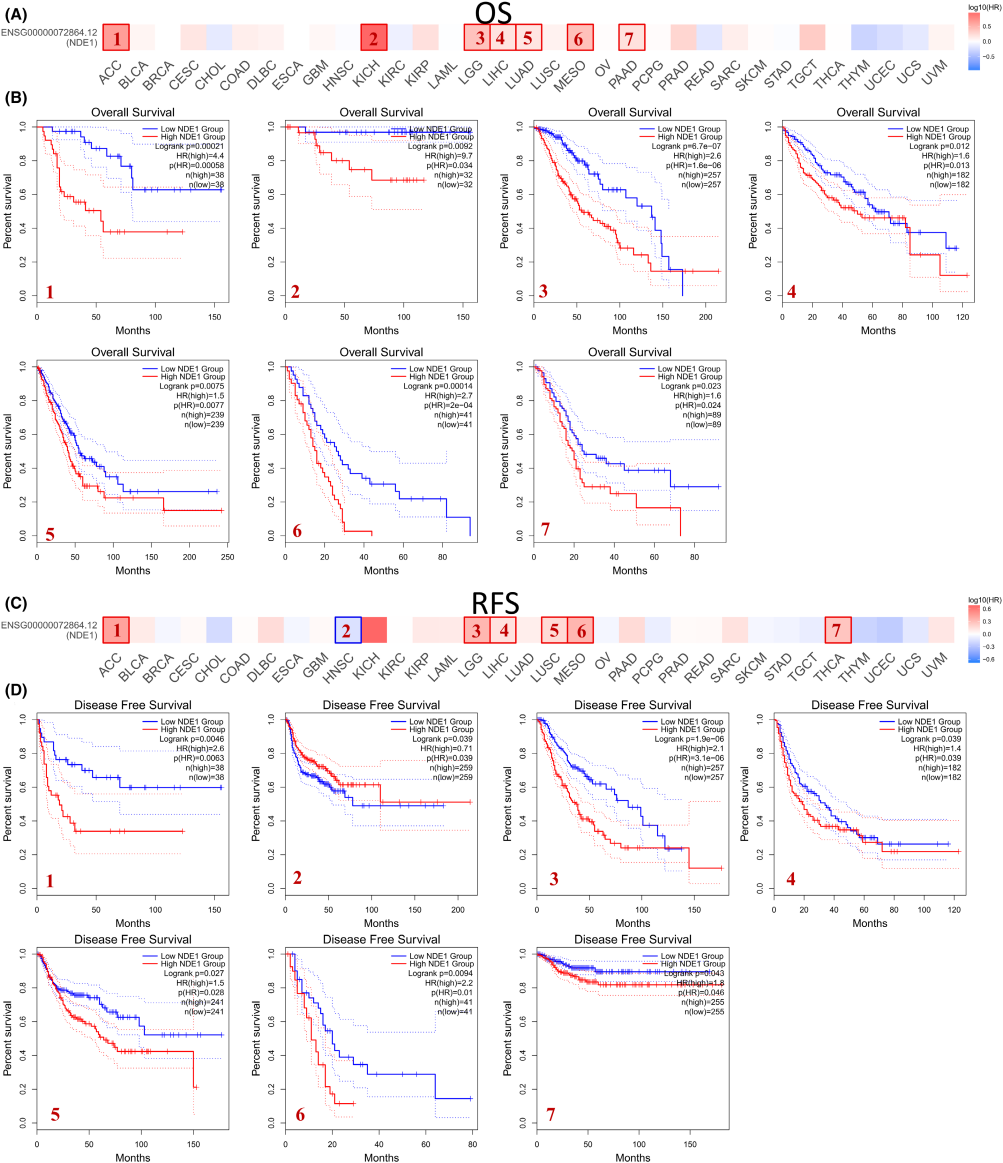
Association of NDE1 with pan‐cancer prognosis. (A, B) The correlation between NDE1 expression and overall survival (OS) in various cancers in TCGA database; (C, D) The correlation between NDE1 expression and recurrence free survival (RFS) in various cancers in TCGA database.

According to the median of NDE1 expression, the tumour samples were divided into high‐ and low‐expression groups using the GEPIA2 database. A survival curve was then created to compare the overall survival (OS) and disease‐free survival (DFS) rates. Low NDE1 groups were observed to have statistically superior OS compared to high NDE1 groups in the following studies: ACC, KICH, LGG, LIHC, LUAD, MESO and PAAD (Figure 2B). The DFS of patients with low NDE1 expression was also superior to that of patients with high NDE1 expression in the ACC, LGG, LIHC, LUSC, MESO and THCA, whereas the DFS of patients with high NDE1 expression was superior to that of patients with low NDE1 expression in the HNSC. Furthermore, we noted that there was also a trend that lower NDE1 expression levels were associated with longer OS or DFS in patients with BLCA, CESC, COAD, DLBC, KICH, KIRP, LUSC, PAAD, SARC and UVM, although no statistical significance was observed (Figure S1). Then, the coxph function of the R package survival (version 3.2–7) was used to establish Cox proportional hazards regression mode to analyse the relationship between gene expression and OS in each tumour. The findings indicated that elevated expression levels in 10 distinct tumour types (GBMLGG, LGG, LAML, KIPAN, LIHC, BLCA, MESO, PAAD, LAML, ACC and KICH) were associated with unfavourable prognosis. Conversely, reduced expression levels in two specific tumour types (ALL, READ) were linked to bad prognosis(Figure S2A). Additionally, when the progression‐free interval and NDE1 expression were examined, it was shown that the high levels of NDE1 expression in eight different tumour types (GBMLGG, LGG, LIHC, BLCA, MESO, PAAD, ACC and KICH) were associated with a poor prognosis (Figure S2B). In summary, increased NDE1 expression is linked to a poor prognosis in the majority of tumours.

### 3.3 NDE1's diagnostic utility in a variety of malignancies

Using the R package ‘pROC’ (v1.17.0.1), ROC curves of NDE1 in various malignancies may be produced to evaluate the diagnostic usefulness of NDE1 in pan‐cancer. The findings demonstrated that NDE1 was a useful diagnostic tool for a number of malignancies. The diagnostic accuracy is quantified using the area under the curve (AUC). In six malignancies, including CHOL (AUC = 0.997), COAD (AUC = 0.931), COADREAD (AUC = 0.923), HNSC (AUC = 0.939), LUSC (AUC = 0.974) and OSCC (AUC = 0.935), the AUC of NDE1 was larger than 0.7 in 16 different forms of cancer (Figure S3).

### 3.4 NDE1 genetic alteration and DNA methylation in pan‐cancer

**FIGURE 3:**
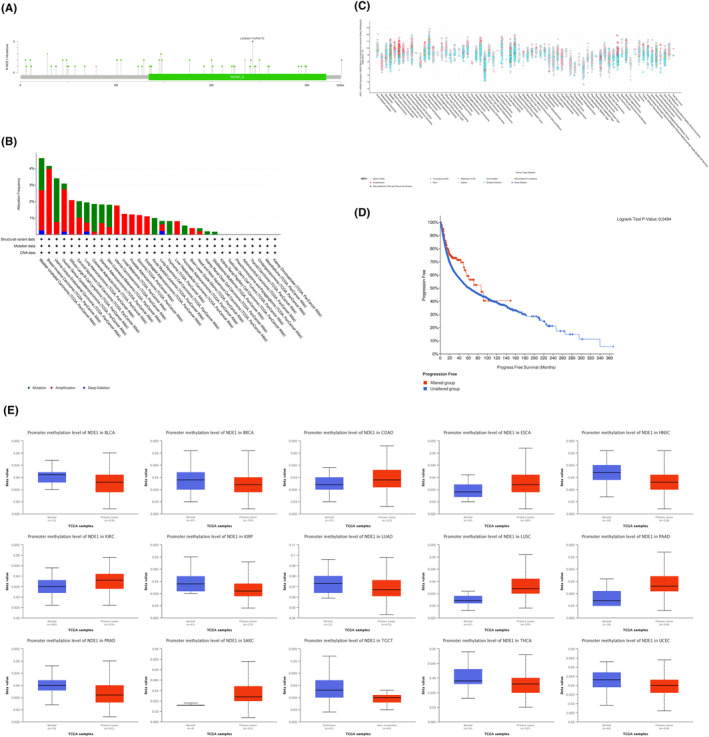
The genetic alteration and DNA modification character of NDE1; (A) The frequency and types of NDE1 somatic mutations in pan‐cancer; (B) Alteration frequency of NDE1 in pan‐cancer; (C) The counts and types of NDE1 mutation in pan‐cancer; (D) Effect of NDE1 genetic alterations on progression‐free survival in patients with cancer; (E) The DNA promotor methylation levels of NDE1 in various cancers and normal tissues in TCGA database.

The location, nature and degree of DNA methylation of NDE1 gene changes may be examined using the cBioPortal platform and the UALCAN web portal. The most vulnerable NDE1 mutation locations are shown in Figure 3A. Deep deletions were the sort of genetic mutation that happened the least often in NDE1, as shown in Figure 3B, happening only in BLCA, OV, LUAD and LUSC. But a number of malignancies often exhibit gene mutations and amplifications. The majority of the NDE1 gene modifications were shallow deletions, according to further investigation of the kinds of mutations (Figure 3C). Furthermore, an examination was conducted to explore the correlation between genetic alterations and the prognostic outcomes across various forms of cancer. The results of the study revealed that genetic modifications significantly enhanced the prognosis‐free survival of those diagnosed with cancer (Figure 3D). Additionally, growing research in recent years has shown a strong link between DNA methylation and the development of cancer.^17^ DNA methylation may play a significant role in the epigenetic regulation of cancer cell proliferation, invasion, differentiation and death. The methylation status of several genes has emerged as a crucial prognostic indicator for tumour prognosis.^18^ DNA methylation may either promote or prevent tumour development. Using the UALCAN web portal, the methylation status of the NDE1 promoter region may be examined. The NDE1 promoter methylation level was considerably greater in BLCA, BRCA, HNSC, KIRP, LUAD, PRAD, TGCT, THCA and UCEC than it was in normal tissues. The NDE1 promoter's methylation level was considerably lower in COAD, ESCA, KIRC, LUSC, PAAD and SARC than it was in normal tissues (Figure 3E). More research is required to pinpoint the specific impact of DNA methylation on NDE1 expression levels in the aforementioned malignancies; however, there may be a link between methylation levels and NDE1 expression levels.

### 3.5 Examination of the relationship between NDE1 and the proteins that bind to it

**FIGURE 4:**
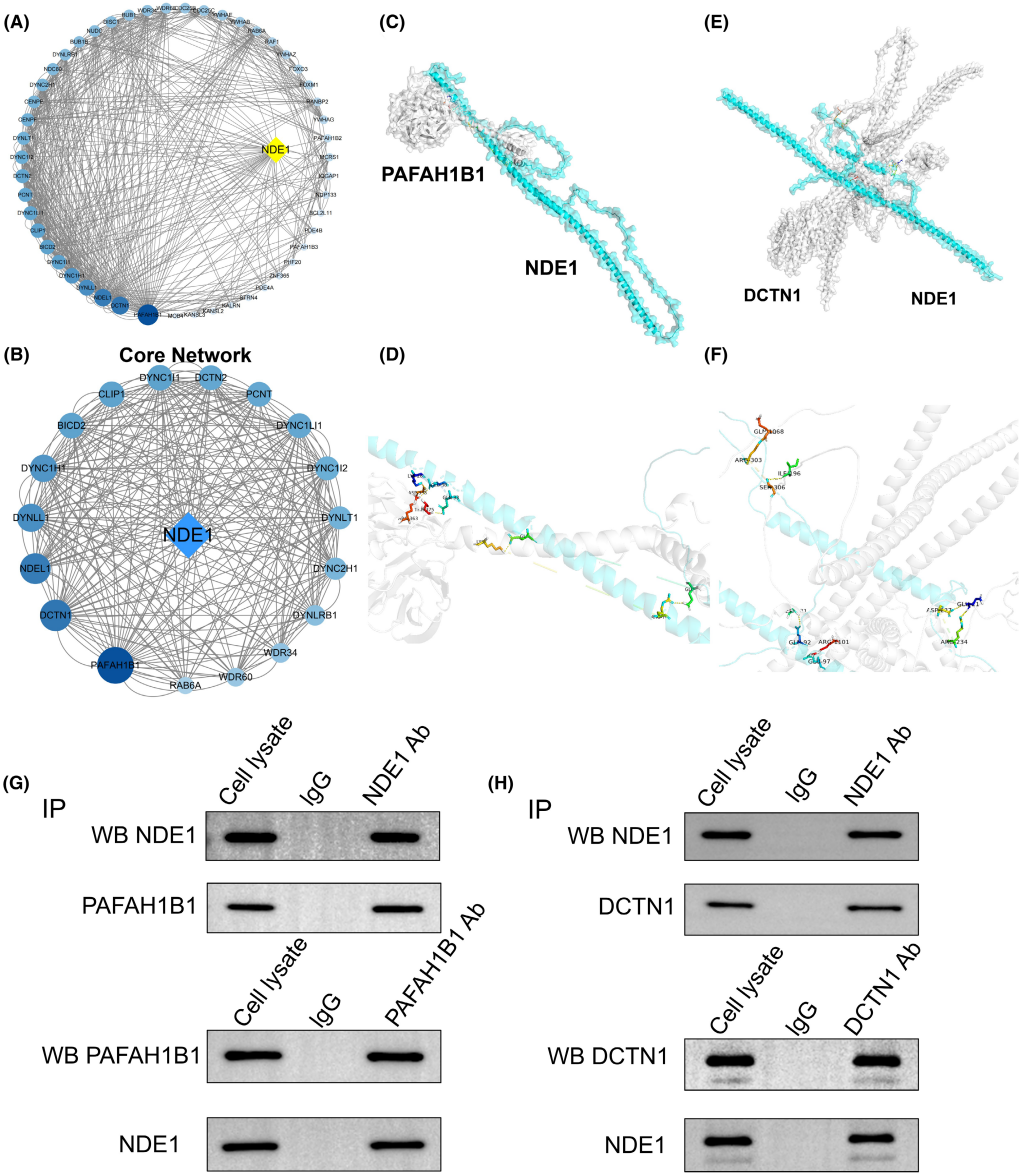
Analysis of NDE1 and its binding proteins. (A) Interaction network between NDE1 and its binding proteins; (B) Core interaction network of NDE1 and its binding proteins; (C–F) Peptide chain structures and interacting binding sites of NDE1, PAFAH1B1 and DCTN1; (G) Co‐immunoprecipitation detected the interaction between NDE1 and PAFAN1B1 in T24 cells. The interaction between NDE1 and PAFAN1B1 was confirmed by western blot. (H) Co‐immunoprecipitation detected the interaction between NDE1 and DCTN1 in T24 cells. The interaction between NDE1 and DCTN1 was confirmed by western blot.

Through STRING analysis, we identified a number of proteins that are closely connected to the NDE1 interaction and that not only interact with NDE1 directly but also, to varying degrees, with other proteins. The 50 proteins that interact with NDE1 are shown in Figure 1, along with the connections between them based on how much they interact with one another (Figure 4A). A core network of NDE1 protein interactions was built using the top 18 proteins (Figure 4B). Finally, we thoroughly examined the interactions between DCTN1 and NDE1 and PAFAH1B1 with NDE1. NDE1, PAFAH1B1 and DCTN1 peptide chain structures were predicted, and binding sites for the interactions were found (Figure 4C,D). To verify the above results, co‐immunoprecipitation experiments were performed. Co‐immunoprecipitation assays demonstrated that NDE1 retrieved PAFAH1B1 and PAFAH1B1 retrieved NDE1, which suggests that NDE1 and PAFAH1B1 physically bind with each other (Figure 4G). Similarly, NDE1 and DCTN1 physically bind with each other, too (Figure 4H). A previous study has shown that overexpression of PAFAH1B1 is associated with poor prognosis of lung adenocarcinoma, and the underlying mechanism may be to promote the migration and invasion of lung cancer cells by disrupting the microtubule network.^19^ In addition, there is also a study showing that the expression level of DCTN1 in colorectal cancer tissues is higher than that in adjacent tissues, which may be an oncogene in colorectal cancer.^20^ We also analysed the effects of PAFAH1B1 and DCTN1 on OS in various cancers, and found that the high expression of these two genes is associated with poor prognosis of various cancers (Figure S4). Both PAFAH1B1 and DCTN1 are shown to promote cancer in specific cancers, while NDE1 has mutual binding properties with PAFAH1B1 and DCTN1, respectively. This also seems to be a piece of evidence that NDE1 behaves as an oncogene in pan‐cancer.

### 3.6 The interactions of NDE1 with NDE1 mRNA's m6A modification

**FIGURE 5:**
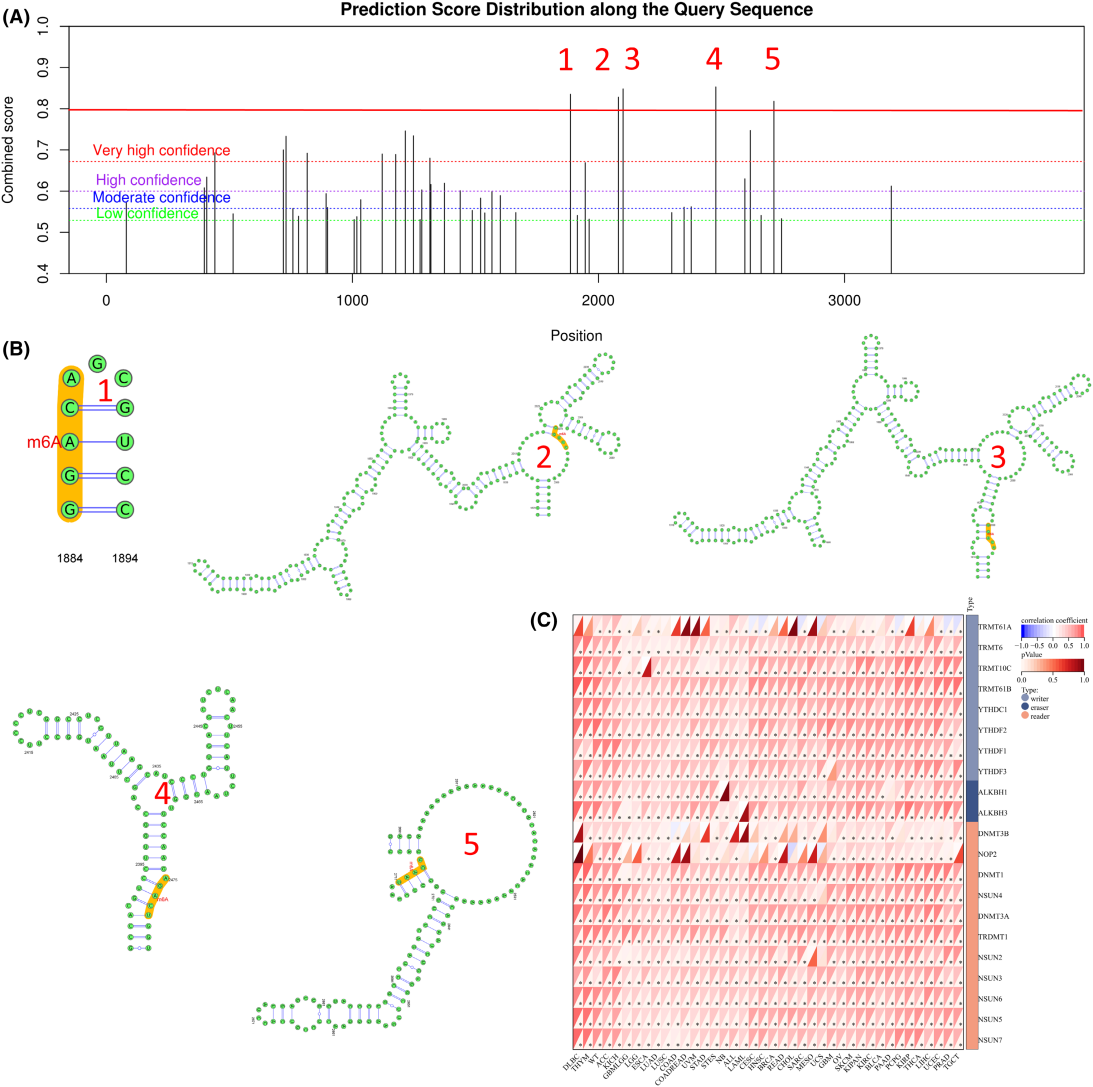
The m6A modification of NDE1 mRNA. (A) Locus of interest; (B) Structure; (C) Related genes. *p < 0.05.

The most frequent internal post‐transcriptional alteration that determines the destiny of a eukaryotic mRNA is called m6A.^21^ The dynamic, reversible alteration of RNA m6A is essential for the development and spread of tumours. Oncogene and tumour suppressor gene m6A alteration research may provide fresh approaches to the detection and therapy of cancer.^22^ The m6A alteration sites of NDE1 were examined via the SRAMP portal. There is a very strong probability that m6A may modify 15 of NDE1's functional regions. The five functional regions with the greatest confidence are shown in Figure 5B. Finally, we further analysed the m6A modifier genes of NDE1 mRNA (Figure 5C). These findings imply that m6A alterations may play a role in how NDE1 affects tumours.

### 3.7 Genomic heterogeneity, immune checkpoint gene analysis and immune infiltration analysis

**FIGURE 6:**
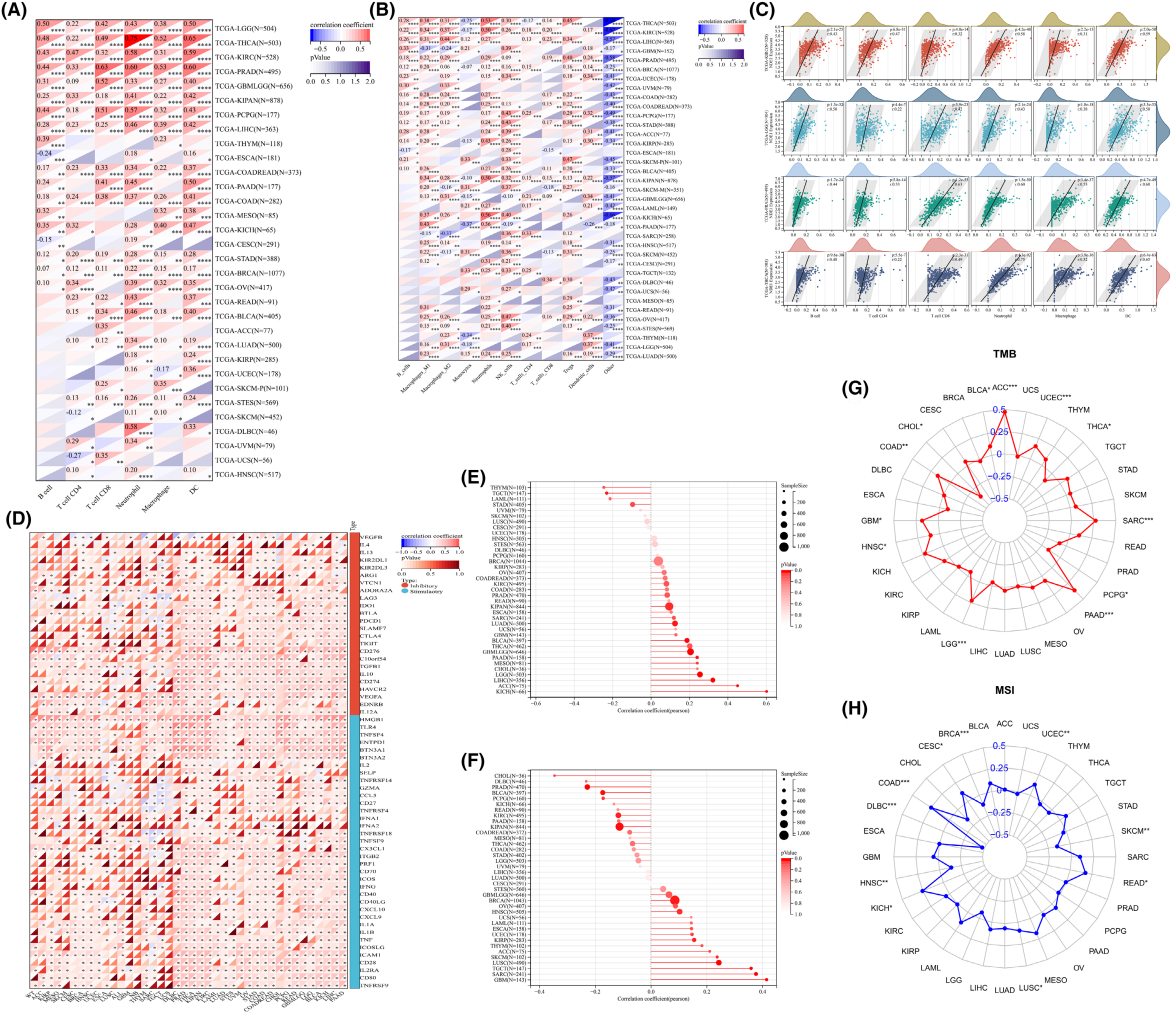
Immunological association analysis of NDE1. (A) The correlation between NDE1 and immune cell infiltration was analysed by TIMER2 database; (B) The correlation between NDE1 and immune cell infiltration was analysed by using QUANTISEQ method; (C) The correlation between immune infiltration and NDE1 in KIRC, LGG, PRAD and THCA; (D) Association of NDE1 with dozens of immune checkpoint genes in pan‐cancer; (E) The correlation between HRD and NDE1 in pan‐cancer; (F) The correlation between NDE1 and tumour extent in pan‐cancers; (G) The correlation between NDE1 and TMB in pan‐cancers; (H) The correlation between NDE1 and MSI in pan‐cancers. *p < 0.05; **p < 0.01; ***p < 0.001 and ****p < 0.0001.

We used the TIMER2 database and the QUANTISEQ method, respectively, to conduct a correlation study between immune cell infiltration and NDE1 expression in pan‐cancer to examine the connection between the two. NDE1 expression was shown to be strongly connected with the infiltration of B cells, CD8+ T cells, CD4+ T cells, neutrophils, macrophages and other immune cells in the majority of malignancies, according to TIMER2 database analysis (Figure 6A). According to the analysis's findings utilising the QUANTISEQ method, NDE1 expression levels were favourably connected with immune cell infiltration in most malignancies, including those of NK cells, regulatory T cells (Tregs), neutrophils, and macrophage‐M1 and M2 cells (Figure 6B). Additionally, we visualised the connection between immune infiltration and NDE1 in different tumours in the TIMER2 database using scatter plots. The four malignancies in the TIMER2 database that had the strongest correlation with immune cell infiltration are shown in Figure 6C. The prevalence and prognosis of cancer are impacted by the immunosuppressive effects of several immune checkpoint pathways.^23^ The identification of novel immunotherapy pathways may greatly benefit from research on the relationship between immune checkpoint genes and NDE1. Given that NDE1 significantly positively correlates with almost all of the known immune checkpoint genes, it is clear that NDE1 is connected to immunity against cancer tumours (Figure 6D). The presence of homologous recombination deficit (HRD), which may result in precise, quantitative and persistent genomic alterations, is a critical marker of therapy choices and prognosis in a number of tumour types. Clinical research has shown a strong correlation between HRD status and susceptibility to platinum‐based chemotherapeutic medications and PARP inhibitors.^24^ In 11 tumours, we found a substantial correlation between HRD and NDE1, with 10 tumours (GBMLGG, LGG, LUAD, KIPAN, LIHC, MESO, PAAD, BLCA, ACC and KICH) showing a significant positive association and 1 tumour (TGCT) showing a significant negative association (Figure 6E). Along with tumour cells, non‐tumour cells such as immune cells, stromal cells and stromal cells in tumour tissues also play a role in the emergence and growth of tumours.^25^ In 15 tumours, a strong correlation between NDE1 and tumour purity was discovered. They included nine tumours (GBM, BRCA, SARC, KIRP, UCEC, HNSC, LUSC, TGCT and SKCM) with substantial positive associations and 6 tumours (KIPAN, PRAD, KIRC, PCPG, BLCA and CHOL) with significant negative correlations (Figure 6F). Microsatellite instability (MSI) and tumour mutation burden (TMB) are two clinically significant immune indicators that are strongly linked to tumour immunotherapy.^26^ We used the TMB function in the R package maftools (version 2.8.05) to determine the tumour's total metabolic burden (TMB), and we then looked at the relationship between TMB and NDE1 expression level. ACC, BLCA, COAD, GBM, HNSC, LGG, PAAD, SARC and UCEC all showed substantial positive associations, while CHOL, PCPG and THCA showed significant negative correlations (Figure 6G). NDE1 expression level showed a substantial positive link with MSI in seven tumours and a significant negative correlation with MSI in four cancers (Figure 6H). As a result, NDE1 may influence the epigenetic state of tumours to control tumour immunity.

### 3.8 NDE1 and cancer immunological and molecular subtypes: Correlation analysis

**FIGURE 7:**
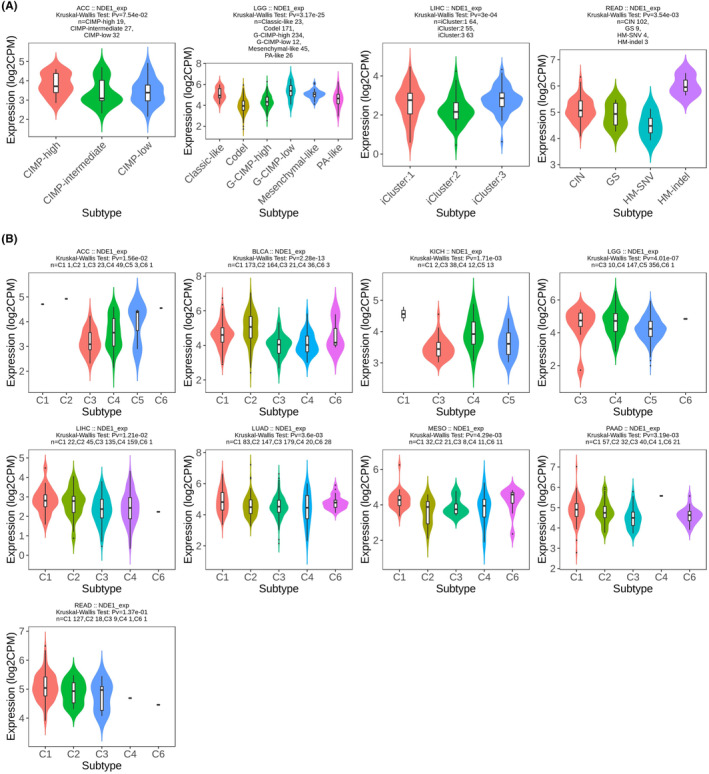
The analysis of NDE1 expression in different immune and molecular subtypes across cancers. (A) The analysis of NDE1 expression in different molecular subtypes in 4 cancer; (B) The analysis of NDE1 expression in different immune subtypes in 10 cancers. C1 (wound healing); C2 (IFN‐gamma dominant); C3 (inflammatory); C4 (lymphocyte depleted); C5 (immunologically quiet); C6 (TGF‐b dominant).

Using the TISIDB portal, the link between NDE1 expression level and cancer immune subtypes and molecular subtypes was examined. The findings demonstrated a correlation between the degree of NDE1 expression and the immunological subtypes of 30 tumours and the molecular subtypes of 17 tumours. According to our earlier findings, the expression level of NDE1 in the TCGA database was related to the overall survival (OS) of 12 different cancer types (ACC, BLCA, GBMLGG, KICH, KIPAN, LAML, LGG, LIHC, LUAD, MESO, PAAD and READ). In nine of these 12 malignancies (ACC, BLCA, KICH, LGG, LIHC, LUAD, MESO, PAAD and READ), NDE1 was connected to immunological subtypes (Figure 7B). In addition, NDE1 was linked to molecular subtypes in 4 of the 12 malignancies (ACC, LGG, LIHC and READ) (Figure 7A). The additional graphic illustrates how NDE1 and other tumours' immunological and molecular subtypes are related to one another (Figures S5 and S6).

### 3.9 Analysis of NDE1‐related genes in pan‐cancer

**FIGURE 8:**
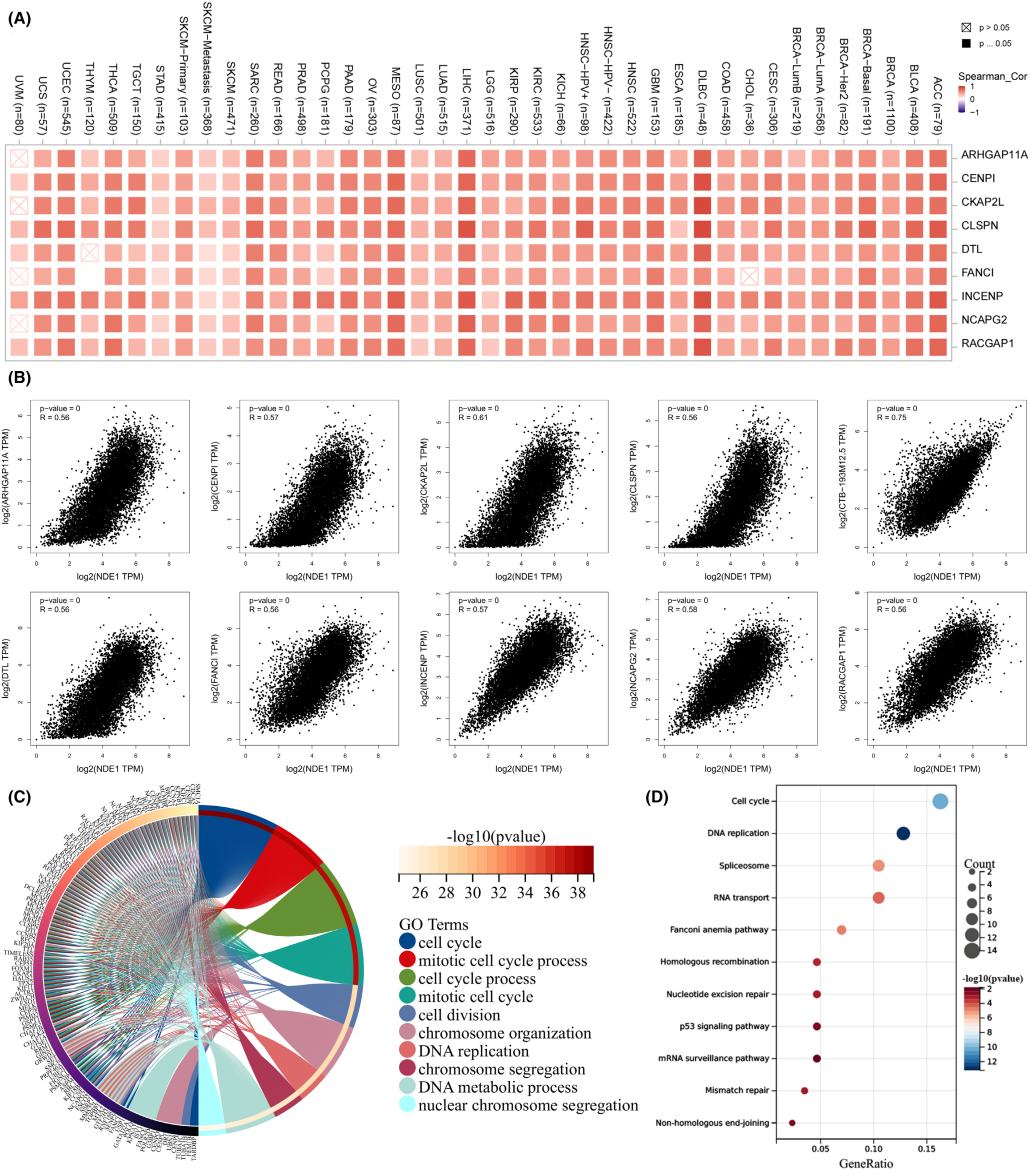
Analysis of NDE1‐related genes in pan‐cancer. (A) NDE1‐related genes in 33 cancers predicted using TIMER2 database; (B) NDE1‐related genes in 33 cancers predicted using GEPIA2 database; (C) GO enrichment analysis of NDE1‐related genes; (D) KEGG enrichment analysis of NDE1‐related genes.

At the transcriptional or other regulatory level, some genes have a reciprocal regulatory association, whereby their expression levels demonstrate a notable connection, either positive or negative. The term ‘related genes of NDE1’ is used to denote genes that have a substantial correlation in their expression levels with NDE1. The investigation of NDE1's main functions and associated pathways may be conducted via the use of pathway and function enrichment analyses on the genes that are closely linked to it. NDE1 showed the strongest association with the following 10 genes: CTB‐193M12.5 (*R* = 0.75), CKAP2L (*R* = 0.61), NCAPG2 (*R* = 0.58), CENPI (*R* = 0.57), NCENP (*R* = 0.57), ARHGAP11A (*R* = 0.56), CLSPN CENPI (*R* = 0.56), DTL (*R* = 0.56), and FANCI (*R* = 0.56) (Figure 8B). Subsequently, the TIMER2 database was used to validate the prognostic outcomes obtained from the GEPIA2 database. With the exception of CTB‐193M12.5, which was not annotated in the TIMER2 database, the other genes were confirmed to be strongly associated with NDE1 (Figure 8A). We conducted GO‐BP and KEGG enrichment analyses of NDE1‐related genes in order to investigate the role of NDE1 in pan‐cancer. According to the results of the GO analysis, the genes associated with NDE1 were mostly concentrated in processes related to cell cycle, cell division, mitotic cell cycle, chromosome segregation, DNA metabolism and nuclear chromosomal segregation (Figure 8C). Therefore, it is hypothesised that NDE1 may control the growth of cancer by influencing the processes of cell division and proliferation. Our hypothesis was further supported by a KEGG enrichment analysis of genes associated with NDE1. Our theory was further supported by KEGG enrichment data, which revealed that NDE1‐related genes were mostly enriched in cell cycle, DNA replication, spliceosome, RNA transport, homologous recombination, mismatch repair, the P53 signalling pathway, etc. (Figure 8D).

We obtained the GSEA database and ran KEGG and GO enrichment analyses of NDE1 to learn more about the role that NDE1 plays in the immune regulation of cancer. According to enrichment findings, NDE1 supports antigen presentation and processing, NK cell‐mediated cytotoxicity and immune effector process control in CHOL (Figure S7A). When NDE1 expression was low compared to high, it was found that the cytokine–cytokine receptor interaction, cytokine‐mediated signalling pathway, monocyte differentiation, positive regulation of cytokine production and regulation of lymphocyte activation were all more active in GBM (Figure S7B). In both KIRC and LGG, NDE1 is connected to cytokine‐related immunity. Additionally, NDE1 may have a role in the activation of the immune system and the modulation of the adaptive immunological response in LGG, as well as the regulation of lymphocyte activation in KIRC (Figure S7C,D). In PRAD, NDE1 was enriched in the control of lymphocyte activation, immunological response‐related cell activation and immune response‐related cell activation (Figure S7E). NDE1 was more abundant in SARC when monocytes and leukocyte‐mediated immunity were differentiating (Figure S7F). Adaptive immune response, monocyte differentiation and cytokine–cytokine receptor interaction were all enriched in NDE1 in SKCM (Figure S7G). The control of lymphocyte activation, the T‐cell receptor signalling pathway and the regulation of lymphocyte activation were all enriched in NDE1 in THCA (Figure S7H). Together, NDE1 may have a role in the control of the immune system in cancer.

### 3.10 Analysis of immunotherapy

We conducted a study using the TISMO database to determine whether the expression level of NDE1 has an effect on tumour immunotherapy. Our data show that NDE1 has a significant predictive value across many mouse immunotherapy groups. Our analysis demonstrates the significant predictive power of NDE1 in multiple murine immunotherapy cohorts. Specifically, NDE1 in MC38_GSE172162_antiPD1, T11_GSE124821_Apobec_day3_antiCTLA4&antiPD1, T11_GSE124821_Apobec_day7_antiCTLA4&antiPD1, YTN1 The expression level of NDE1 in the response group was significantly lower than that in the other groups in the four immunotherapy cohorts of 6_GSE146027_day21_antiCTLA4, while the expression level of NDE1 in the response group was significantly higher than that in the other groups in the treatment cohort of EMT6_GSE107801_antiPDL1 (Figure S8A). In addition, we used the TISMO database to examine NDE1 expression levels in cell lines that received different treatments and found that after IFNg treatment, The expression levels of NDE1 in B16_SSG33589424, CT26_RTM28723893 and EMT6_XW33589424 cohorts were significantly lower than those in other treatment groups and baseline. After IFNb treatment, the expression level of NDE1 in the LLC_RTM28723893 cohort was significantly lower than that in the other treatment groups and baseline (Figure S8B). Moreover, we discovered some relevance for NDE1 in predicting the outcome of anti‐PD‐L1 treatment (Figure S8C). In light of the association between immune infiltration, MSI, TMB, tumour purity and NDE1 expression level that we previously examined, we hypothesise that NDE1 may have some bearing on the sensitivity to tumour immunotherapy.

### 3.11 NDE1 expression levels in tumours are regulated by an upstream lncRNA‐miRNA regulatory network

**FIGURE 9:**
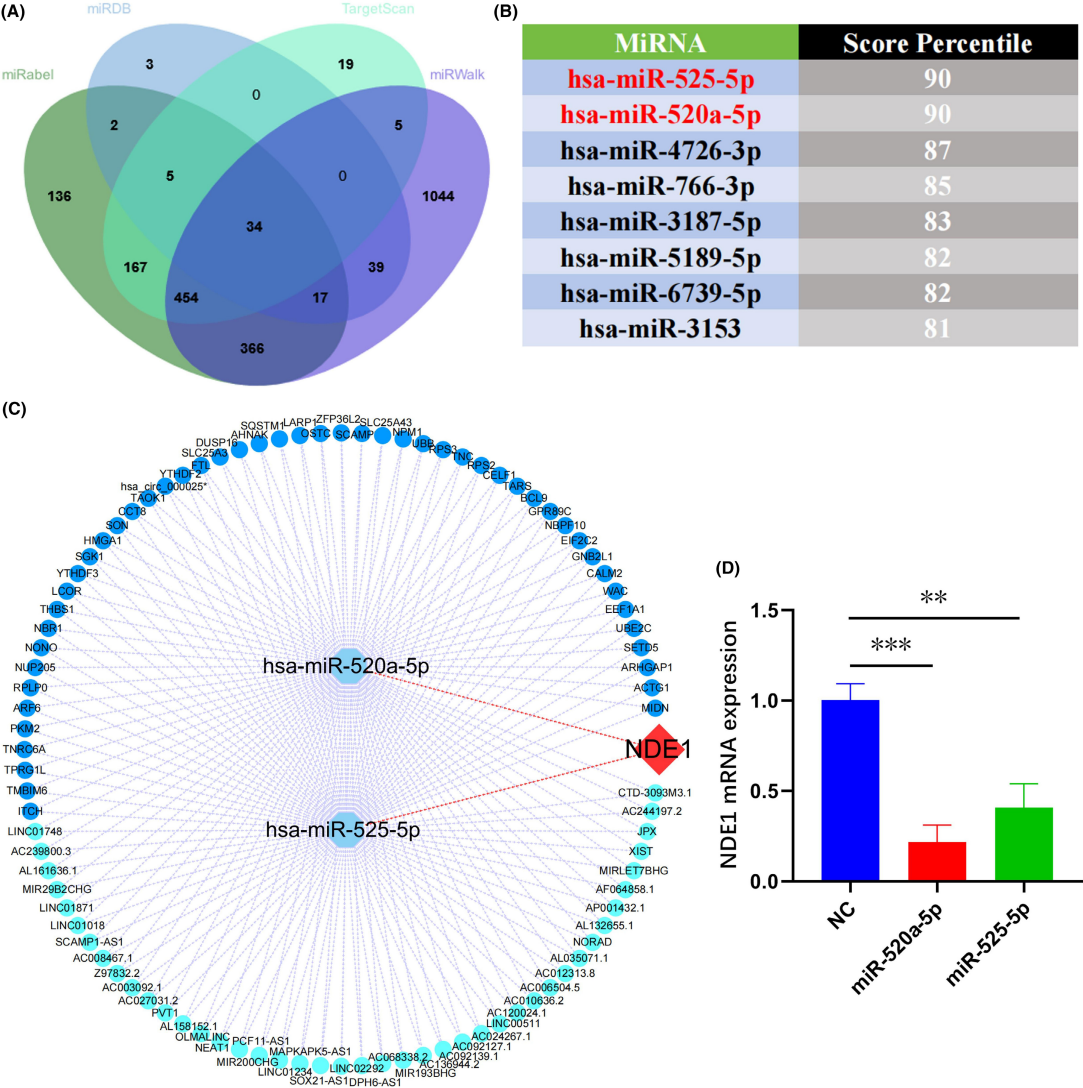
ceRNA network upstream of NDE1. (A) The upstream miRNAs of NDE1 were predicted by miRWalk, miRDB, Targetscan and miRabel databases, and the intersection was taken (34 intersection miRNAs). (B) The top 8 miRNAs targeting NDE1 were displayed in Targetscan database. (C) The top 2 miRNAs targeting NDE1 and the lnRNAs upstream of them. (D) RT‐PCR was used to explore the down‐regulation effect of miR‐520a‐5p and miR‐525‐5p on NDE1 mRNA level.

Numerous studies in recent years have shown that lncrna primarily functions as a competitive endogenous RNA (ceRNA) and indirectly controls the miRNA's downstream pathways by adsorbing the matching miRNA, which has an impact on the development of cancer.^27^, ^28^ To forecast miRNAs upstream of NDE1, we collected data from miRDB, miRWalk, miRabel and TrgetScan. The intersection of the possible miRNAs predicted by the four databases to target NDE1 mRNA led to the identification of 34 miRNAs (Figure 9A). These 34 miRNAs were then scored using TargetScan. The top 8 miRNAs with the highest TargetScan scores were miR‐525‐5p (90), miR‐520a‐5p (90), miR‐4726‐3p (90), miR‐766‐3p (85), miR‐3187‐5p (83), miR‐5189‐5p (82), miR‐6739‐5p (82) and miR‐3153 (81) (Figure 9B). Next, using the lncBase database, the upstream lncRNAs and circRNAs of two miRNAs with scores of 90 were predicted. Finally, using the Cytoscape software, the regulatory network of lncRNA/circRNA‐miRNA‐NDE1 was created (Figure 9C). It is known that miRNAs induce target RNA degradation mainly through incomplete miRNA‐mRNA complementary pairing.^29^ Therefore, we transfected T24 cells with mimics of miR‐520a‐5p and miR‐525‐5p, respectively, and measured the relative expression of NDE1 mRNA by RT‐PCR. The results showed that compared with NC group, transfection of miR‐520a‐5p and miR‐525‐5p significantly down‐regulated NDE1 mRNA level in T24 cells. This also indirectly suggests that miR‐520a‐5p and miR‐525‐5p target NDE1 (Figure 9D).

### 3.12 The role of NDE1 in the proliferation, migration and invasion of bladder cancer cells

**FIGURE 10:**
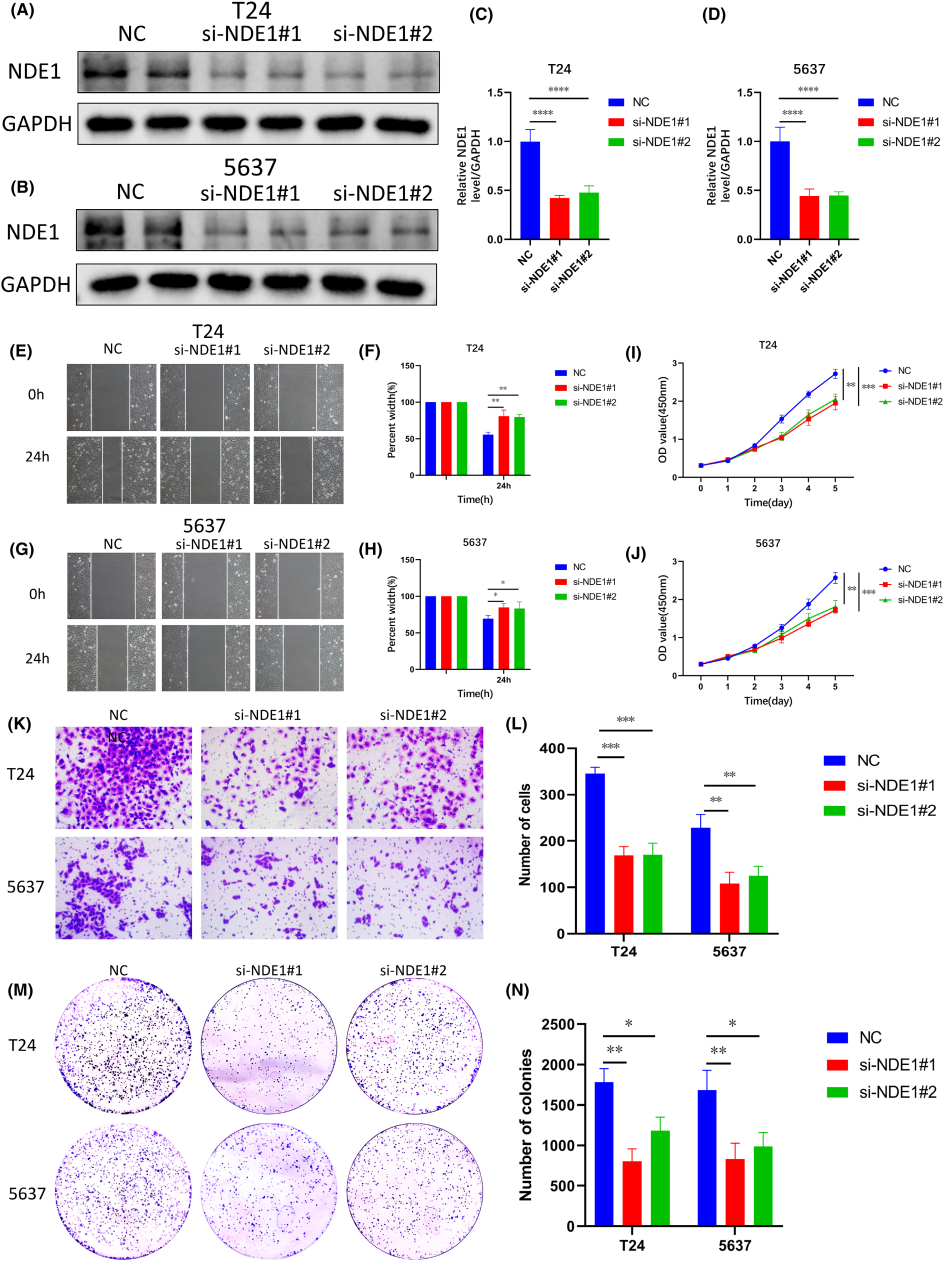
Biological functions of NDE1 in BLCA. (A, C) The knockdown efficiency of NDE1 in T24 cell line was verified by western blot; (B) and (D) NDE1 knockdown efficiency in 5637 cell line was detected by western blot; (E, F) The effect of NDE1 on the migration and invasion of T24 cells was investigated by scratch assay; (G, H) The effect of NDE1 on the migration and invasion of 5637 cells was investigated by scratch assay. (I) To investigate the effect of NDE1 on the proliferation of T24 cells by CCK‐8 assay; (J) CCK‐8 assay was used to investigate the effect of NDE1 on the proliferation of 5637 cells; (K, L) transwell assay was used to investigate the effect of NDE1 on the migration and invasion of T24 and 5637 cells. (M, N) The effect of NDE1 on the proliferation ability of T24 and 5637 cells was explored by colony formation assay. *p < 0.05; **p < 0.01; ***p < 0.001 and ****p < 0.0001.

In light of the aforementioned analysis, it is suggested that NDE1 may exert regulatory control over the proliferation, invasion and migratory processes seen in various malignancies. The expression of NDE1 in BLCA tissues was shown to be considerably higher compared to adjacent tissues. Moreover, there was a strong correlation between the amount of NDE1 expression and the unfavourable prognosis of BLCA. Consequently, it was determined that bladder cancer cells would be used to validate the findings of the bioinformatics research. For the purpose of this study, two conventional bladder cancer (BLCA) cell lines, namely T24 and 5637, were used. We used siRNA to reduce NDE1 expression in cells, and a western blot test was used to confirm the effectiveness of the reduction (Figure 10A–D). The capacity of cells to invade and migrate was then determined using the scratch test, and we discovered that NDE1 knockdown prevented the invasion and migration of T24 and 5637 cells (Figure 10E–H). NDE1 was knocked down, and the CCK‐8 experiment showed that this dramatically slowed cell growth (Figure 10I,J). A transwell assay was used to confirm the impact of NDE1 knockdown on cell invasion and migration (Figure 10K,L). Finally, the colony formation experiment provided further evidence that the capacity of cells to proliferate was considerably decreased by NDE1 knockdown (Figure 10M,N). The functional experiments described above fill a gap in the study of the role of NDE1 in cancer.

**FIGURE 11:**
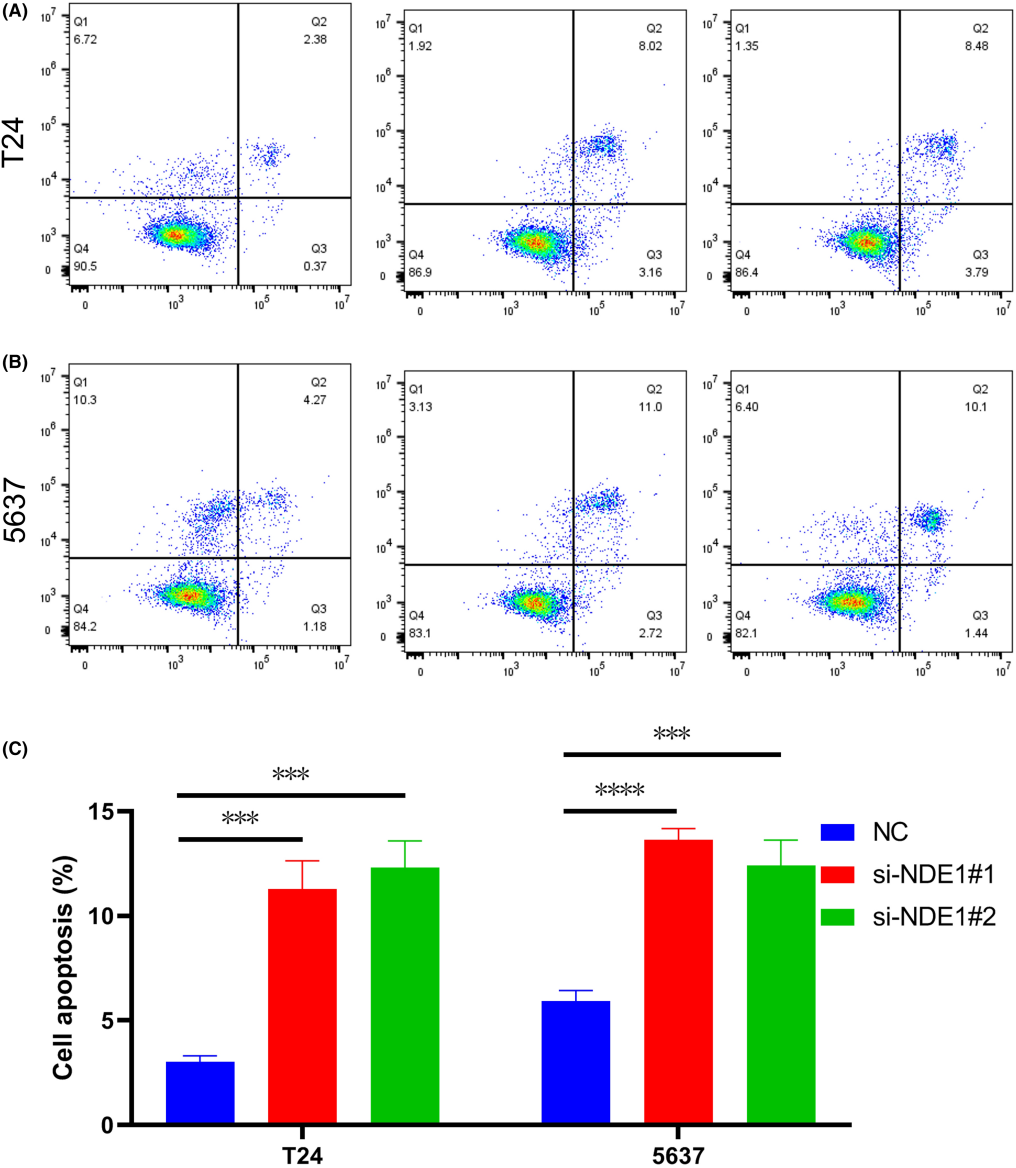
Flow cytometry was used to detect the early apoptosis and late apoptosis cell population in T24 and 5637.

The above studies indicate that NDE1 has a role in promoting the proliferation of bladder cancer cells. This conclusion is also in accordance with the results of the related gene analysis above. Furthermore, it is well known that cancer is not only associated with abnormal activation of cell proliferation, but also often associated with inhibition of apoptosis. Dysregulation of cell proliferation and compensatory inhibition of apoptosis often co‐occur to promote cancer initiation and progression.^30^ Therefore, we explored the effect of knockdown of NDE1 on apoptosis using flow cytometry and showed that knockdown of NDE1 promoted apoptosis in T24 and 5637 cell lines (Figure 11).

## 4 DISCUSSION

Cytoplasmic dynamin‐1 (dynein) is a significant contributor to microtubule transport, playing a crucial role in essential biological processes such as intracellular material transportation and spindle orientation inside eukaryotic organisms.^31^ The material transported by microtubules is very rich; mRNA, organelles and vesicles are just a few of the intracellular molecules that dynein transports. To accurately control cellular processes like mitosis, dynein builds intricate protein complexes with microtubules, molecular motors and other proteins.^32^ The several subunits that make up dynein include the heavy, intermediate, light intermediate and light chains. Dynein cell light intermediate chain 1 (DYNC1LI1) influences the sensitivity of colorectal cancer to radiation and chemotherapy and is linked to pancreatic ductal adenocarcinoma, hepatocellular carcinoma and prostate cancer.^33^ Prostate cancer development has also been shown to be regulated by axonemal dynein heavy chain 8.^34^ In conclusion, dynein may influence cell division, which might contribute to the development of tumours. The dynein adaptor protein Lis1 interacts with NDE1, an essential regulator of dynein, to control mitotic events such as spindle aggregation.^35^ It has also been shown that NDE1 may bind to the cancer‐causing protein p78/MCRS1.^15^ We believed that NDE1 was most likely involved in the formation of cancer since it controls cell division by interacting with dynein and can bind to certain proteins implicated in cancer development. However, there are few or even nonexistent studies on the relationship between NDE1 and tumours. We thus used data mining and tests to investigate the function of NDE1 in pan‐cancers.

In this work, we first examined the amount of NDE1 expression in different malignancies and discovered that NDE1 expression was considerably greater in a number of tumours than in normal tissues. All, BRCA, CESC, ChOL, COAD, COADREAD, ESCA, GBM, GBMLGG, HNSC, KIPAN, KIRC, KIRP, LAML, LGG, LIHC, LUAD, LUSC, OV, PAAD, READ, SKCM, STES, STAD, TGCT and WT were among them. NDE1 was shown to be strongly expressed in BLCA, BRCA, CHOL, COAD, COADREAD, ESAD, ESCA, HNSC, KIRC, KIRP, LIHC, LUAD, LUSC, OSCC, READ and STAD after analysis of matched samples of malignant and paracancerous tissues. Therefore, we make the assumption that NDE1 could function as an oncogene, promoting the development and spread of a range of tumours. Additionally, there was a strong relationship between NDE1 expression and the stages of ACC, KICH, LIHC, PAAD and OV malignancies. NDE1 expression was also substantially linked to poor prognosis in ACC, KICH, LGG, LIHC, LUAD, MESO, LUSC, THCA and PAAD, according to the survival curve. Furthermore, COX regression analysis revealed that increased NDE1 expression negatively impacted the overall survival (OS) of the GBMLGG, LGG, LAML, KIPAN, LIHC, BLCA, MESO, PAAD, LAML, ACC and KICH. It was discovered that NDE1 may be involved in the regulation of cell division, DNA replication, homologous recombination and mismatch repair through KEGG and GO enrichment of NDE1‐related genes, which further indicated that NDE1 may affect the occurrence and development of cancer by controlling the process of cell division. In conclusion, our studies reveal that NDE1 may be an oncogene with potential as a prognostic indicator for cancer.

Methylation has been discovered to control the expression of tumour suppressor or oncogene genes, impacting the development and spread of cancer. Both the gene suppression brought on by hypermethylation of tumour suppressor gene promoters, and the overexpression of oncogenes brought on by genome‐wide hypomethylation contribute to the advancement of cancer.^36^ Additionally, methylation is a significant indicator of several malignancies and has enormous promise for application in the early detection of cancer. For instance, it has been demonstrated that aberrant DNA methylation plays a significant diagnostic role in oesophageal squamous cell carcinoma and that the detection of specific genomic DNA methylation levels in urine can be used to assess the malignancy of bladder cancer and gauge the effectiveness of treatment.^37^, ^38^ In our study, we discovered that NDE1 promoter methylation was significantly increased in BLCA, BRCA, HNSC, KIRP, LUAD, PRAD, TGCT, THCA and UCEC, while NDE1 promoter methylation was significantly decreased in COAD, ESCA, KIRC, LUSC, PAAD and SARC, suggesting that NDE1 promoter methylation may serve as a critical tumour diagnostic marker.

The immune system acts as a watchdog to stop the development of cancer cells. The growth and prognosis of tumours are tightly correlated with the tumour immune microenvironment. Numerous studies have shown that the degree of immune cell infiltration may be able to predict the prognosis of a tumour.^39^, ^40^ Our research revealed a favourable correlation between NDE1 expression levels and immune cell infiltration, including B cells, CD8+ T cells, CD4+ T cells, neutrophils, macrophages, NK cells and regulatory T cells (Tregs), in the majority of malignancies. Programmed cell death is based on immune checkpoints. By bypassing immunological checkpoints, which activate immune cells and cause them to attack cancer cells, several immunotherapy treatments for cancer are made possible.^41^ Our research revealed that NDE1 is strongly linked to tumour mutation burden (TMB), microsatellite instability (MSI), tumour purity and homologous recombination deficit (HRD) in a range of tumours, in addition to immune checkpoint genes in the majority of tumours. Previous research has shown that tumours with elevated TMB and MSI often react better to immunotherapy.^42^ In addition, the lower the purity of the tumour, the more malignant it is and the less sensitive it is to immunotherapy.^43^ Additionally, HRD is a significant marker that may indicate that some immunotherapies are successful in treating malignancies such as ovarian cancer.^44^ As a result, NDE1 has the potential to be a crucial biomarker for immunotherapy and be used to identify individuals who will respond well to it. Additionally, we discovered a link between the effectiveness of immunotherapy and DE1 expression levels. In conclusion, NDE1 has the potential to be a target for immunotherapy since it is associated with the percentage of cancer cells in tumours and other genomic abnormalities, such as spontaneous loss of nucleotides in repetitive DNA strands. These changes determine how sensitive tumour immunotherapy is to treatment.

In summary, our investigation has shown that the expression of NDE1 is significantly elevated in several malignant tumours and is associated with a worse prognosis in numerous cancer types. Moreover, the enrichment of related genes implies that NDE1 is connected to processes like cell division. Multiple studies have shown that a considerable number of tumours have modified NDE1 expression, and many types of cancer also display changed or increased levels of NDE1 methylation. Furthermore, a correlation has been identified between NDE1 and the infiltration of immune cells, the immunological milieu and the sensitivity to immunotherapy. In addition, the results of in vitro experiments demonstrated that the knockdown of NDE1 not only resulted in reduced proliferation, invasion and migration of bladder cancer cells, but also promoted apoptosis. As a result, NDE1 could be an oncogene and have the potential to be a biomarker for the early detection of malignancies that are connected to it, as well as a possible indicator of sensitivity to immunotherapy.

## AUTHOR CONTRIBUTIONS

**Peihan Wang:** Conceptualization (equal); methodology (equal); software (equal); visualization (equal); writing – original draft (lead). **Jinzhuo Ning:** Conceptualization (equal); data curation (lead); writing – review and editing (lead). **Wu Chen:** Conceptualization (equal); methodology (equal); software (equal). **Fan Zou:** Conceptualization (equal); methodology (equal); software (equal). **Weimin Yu:** Investigation (equal); supervision (equal). **Ting Rao:** Investigation (equal); supervision (equal). **Fan Cheng:** Funding acquisition (lead); project administration (lead); supervision (lead).

## FUNDING INFORMATION

This work was funded by the Natural Science Foundation of China (No. 81870471).

## CONFLICT OF INTEREST STATEMENT

The authors declare that there was no conflict of interest.

## Supporting information


Supplementary Figure S1.

Supplementary Figure S2.

Supplementary Figure S3.

Supplementary Figure S4.

Supplementary Figure S5.

Supplementary Figure S6.

Supplementary Figure S7.

Supplementary Figure S8.

## Data Availability

The datasets generated for this study are available on request to the corresponding author.
