# Supplementary figures and images for: Comprehensive analysis indicated that NDE1 is a potential biomarker for pan‐cancer and promotes bladder cancer progression
Source: Cancer Med. 2024 Mar 11;13(5):e6931. doi: 10.1002/cam4.6931 (PMC10926885; doi:10.1002/cam4.6931)

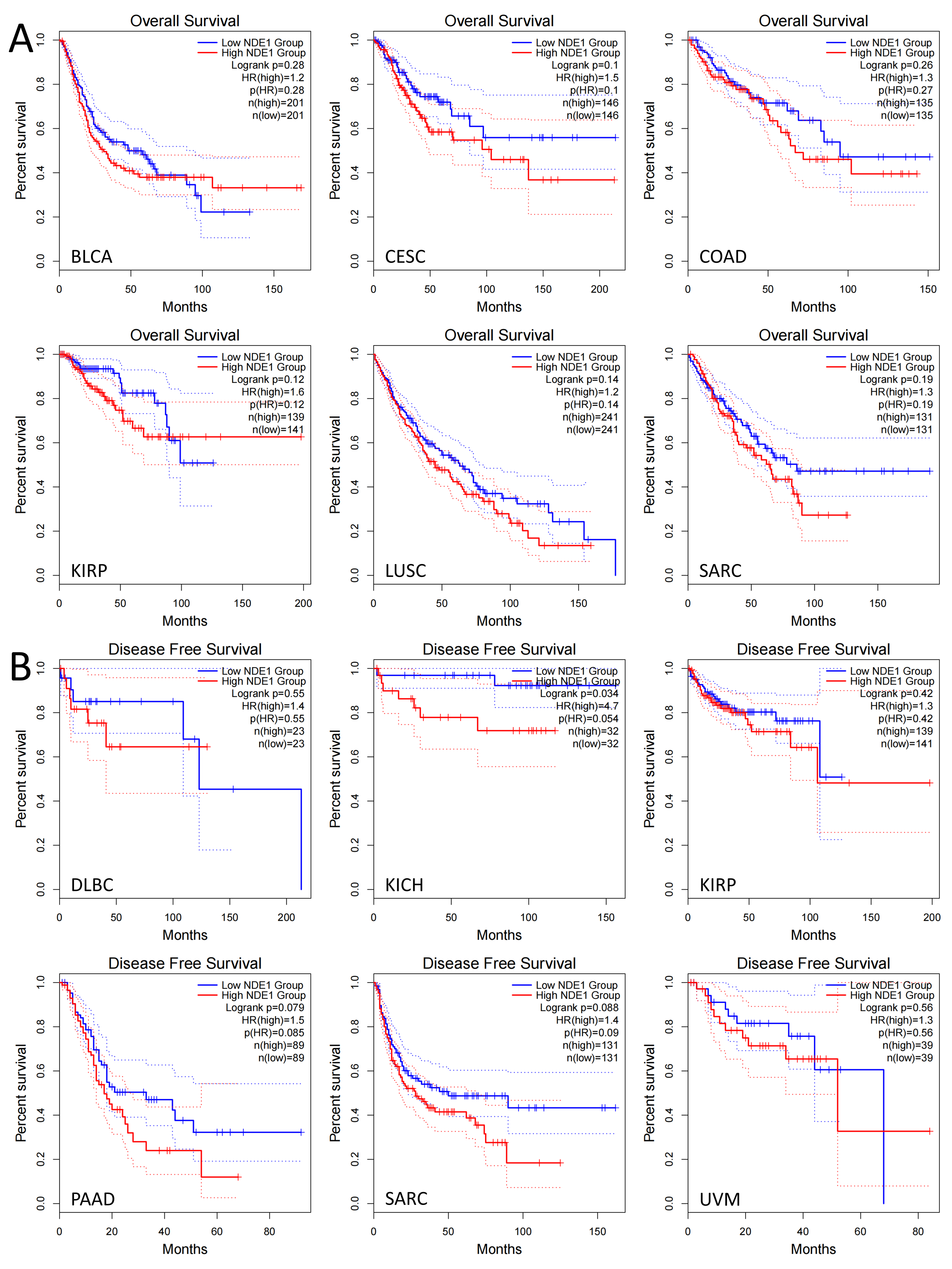

Supplement: Supplementary file 1 — Supplementary Figure S1. [file CAM4-13-e6931-s001.tif]

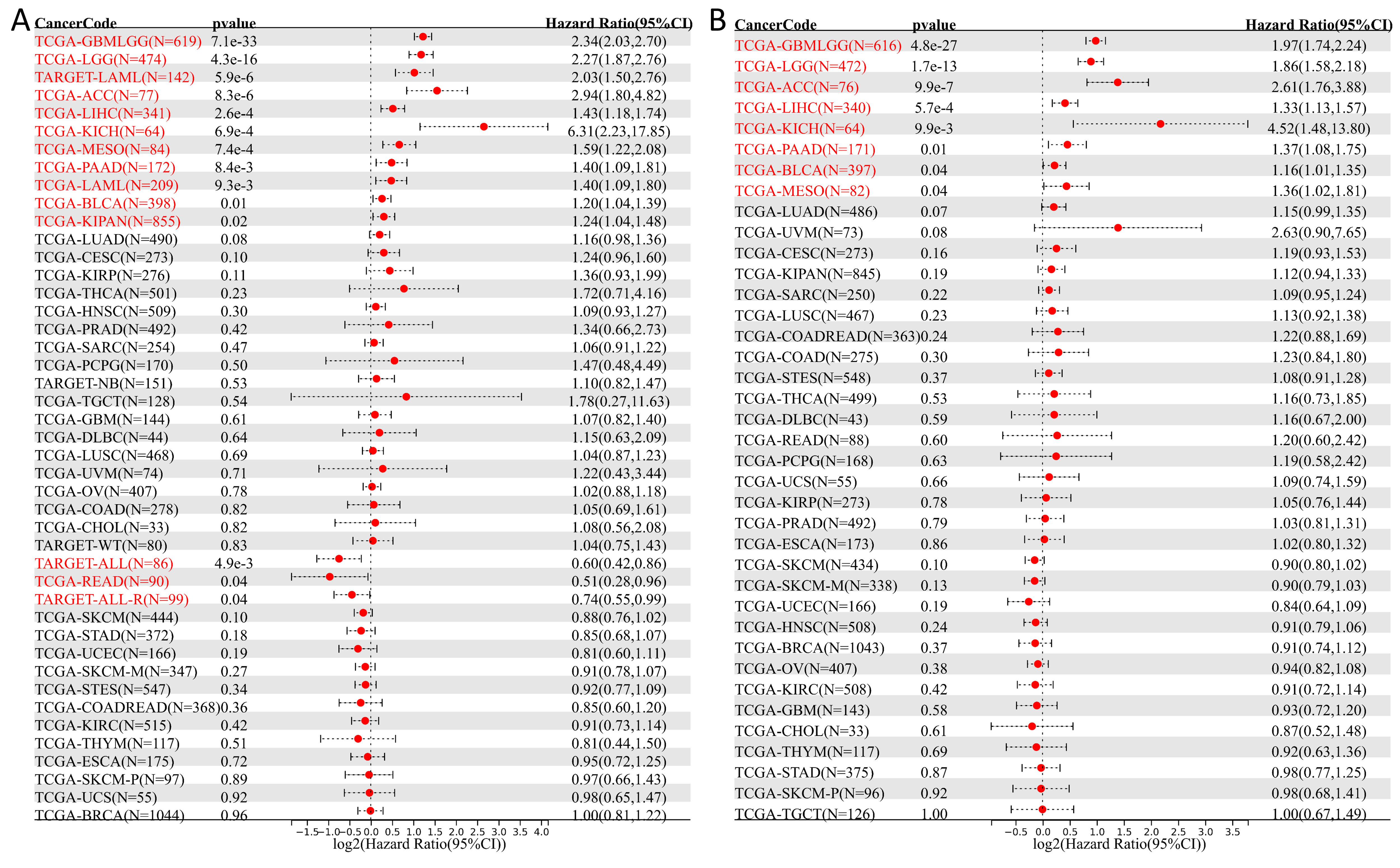

Supplement: Supplementary file 2 — Supplementary Figure S2. [file CAM4-13-e6931-s002.tif]

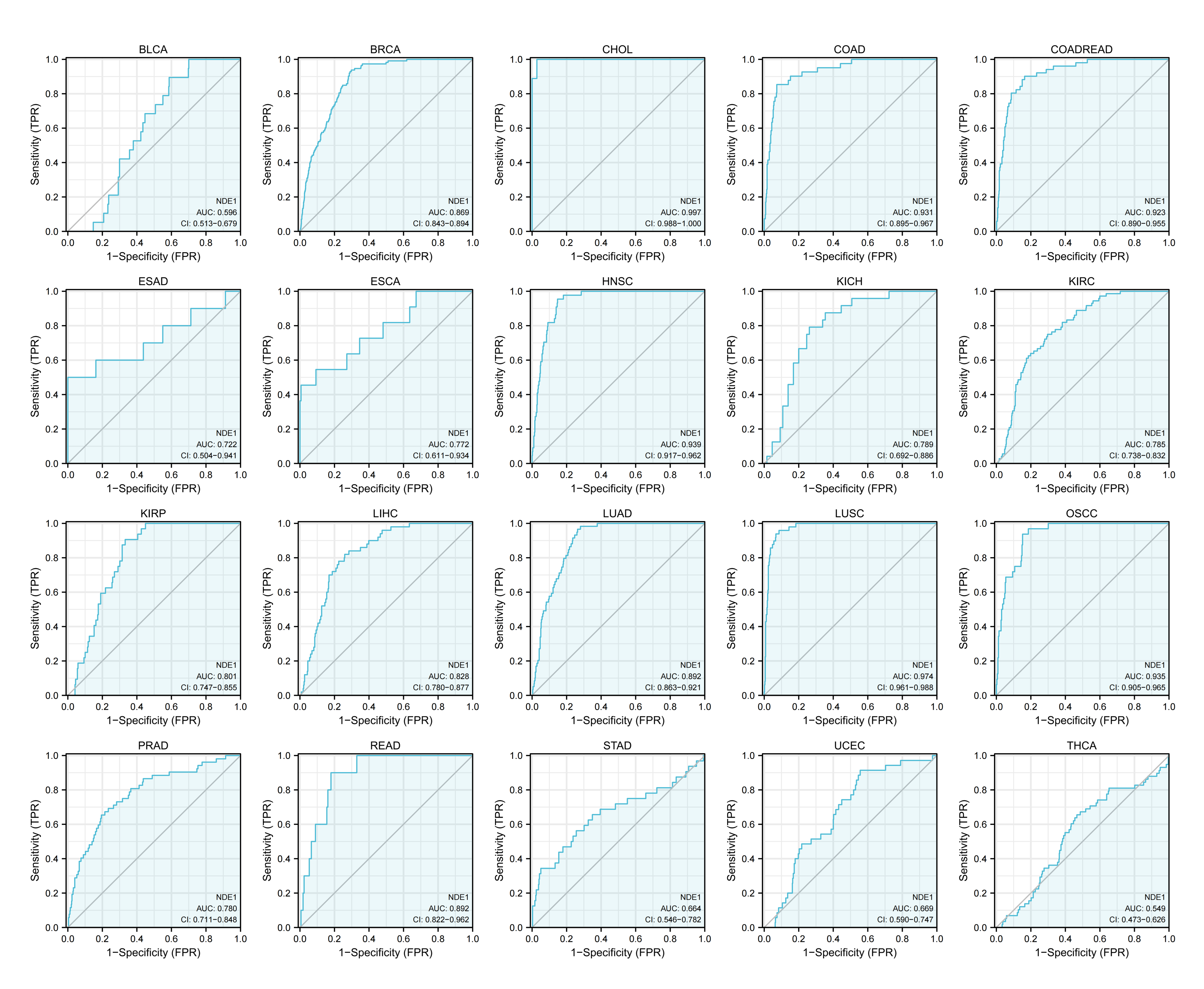

Supplement: Supplementary file 3 — Supplementary Figure S3. [file CAM4-13-e6931-s005.tif]

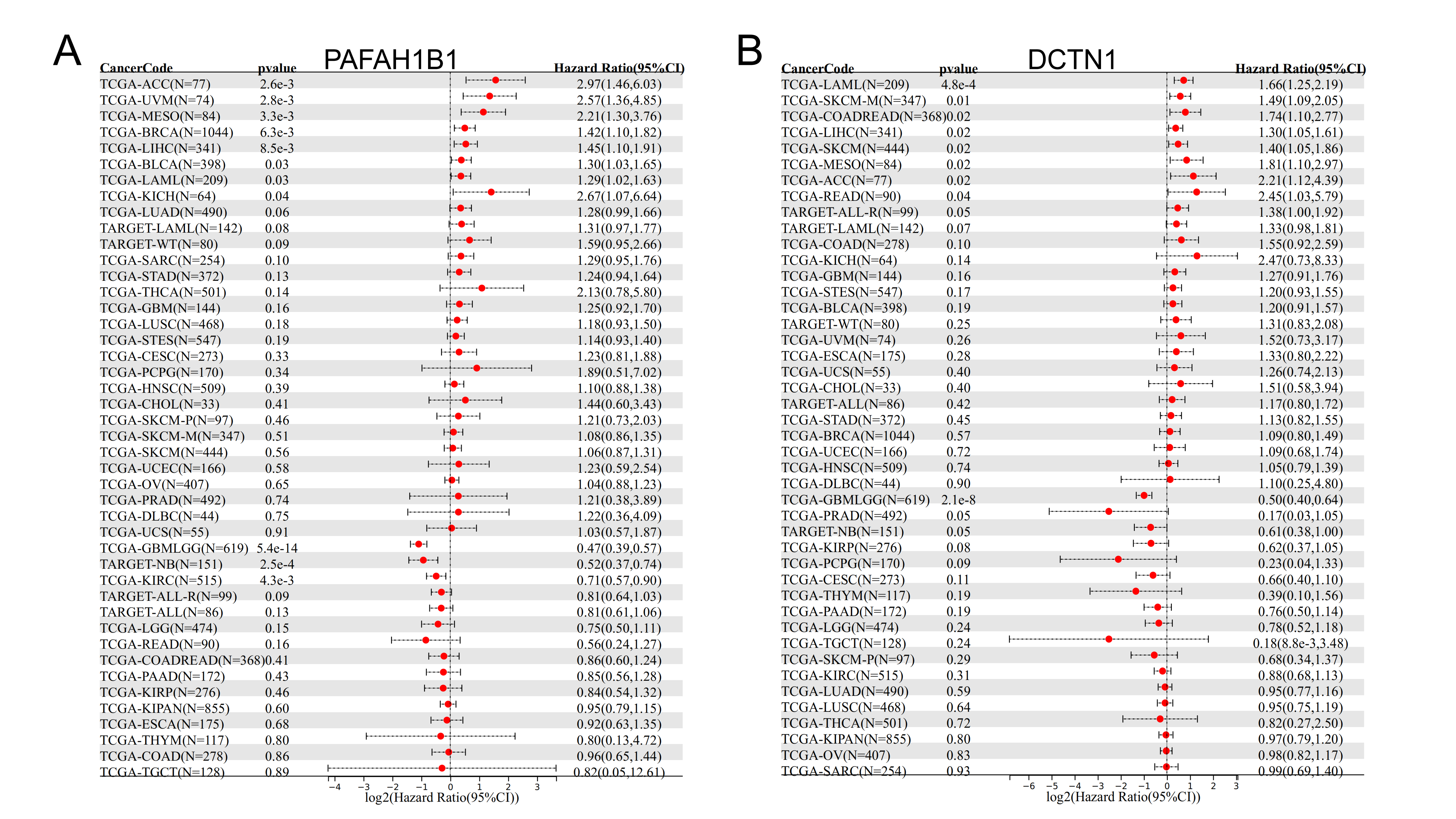

Supplement: Supplementary file 4 — Supplementary Figure S4. [file CAM4-13-e6931-s003.tif]

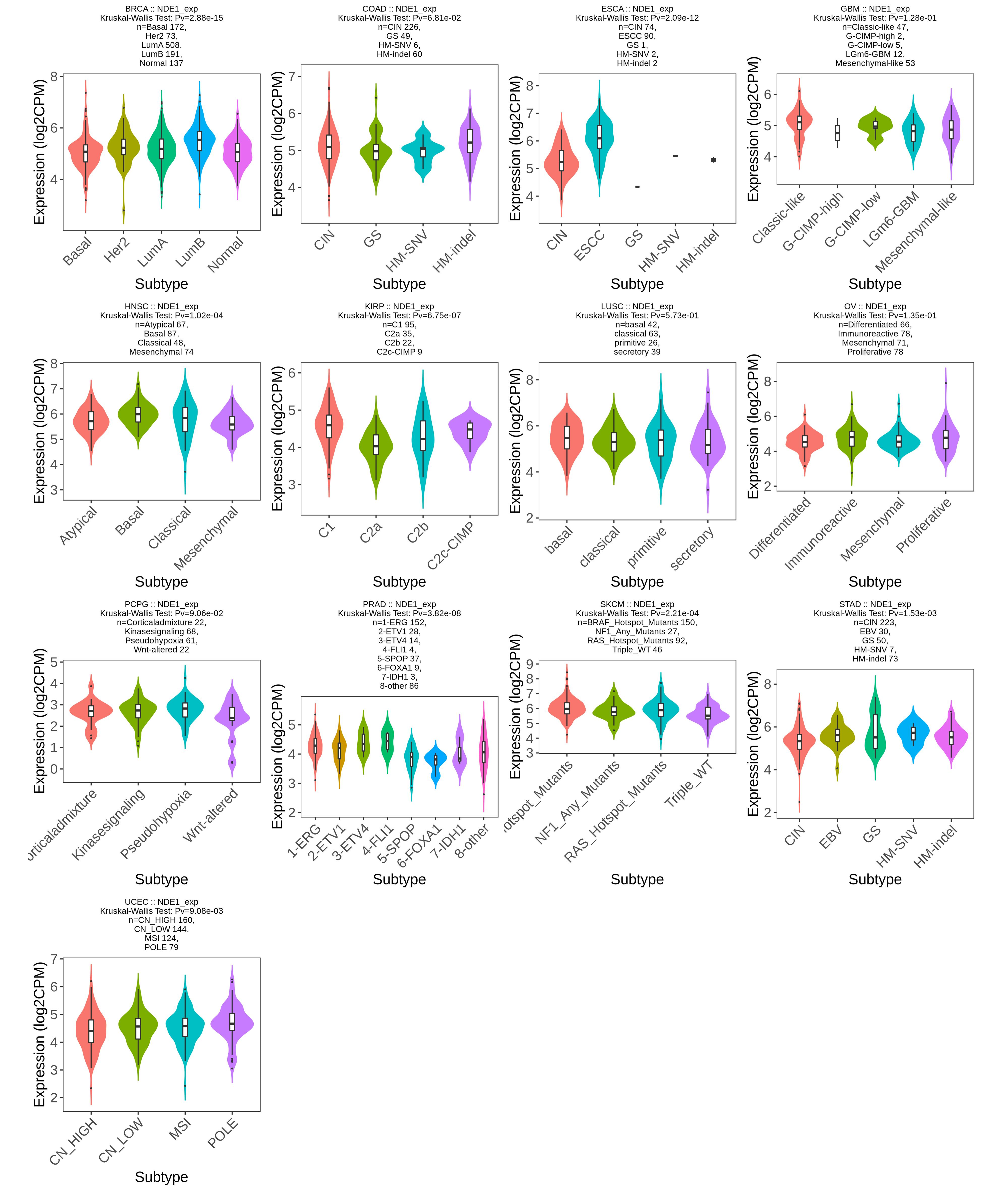

Supplement: Supplementary file 5 — Supplementary Figure S5. [file CAM4-13-e6931-s007.tif]

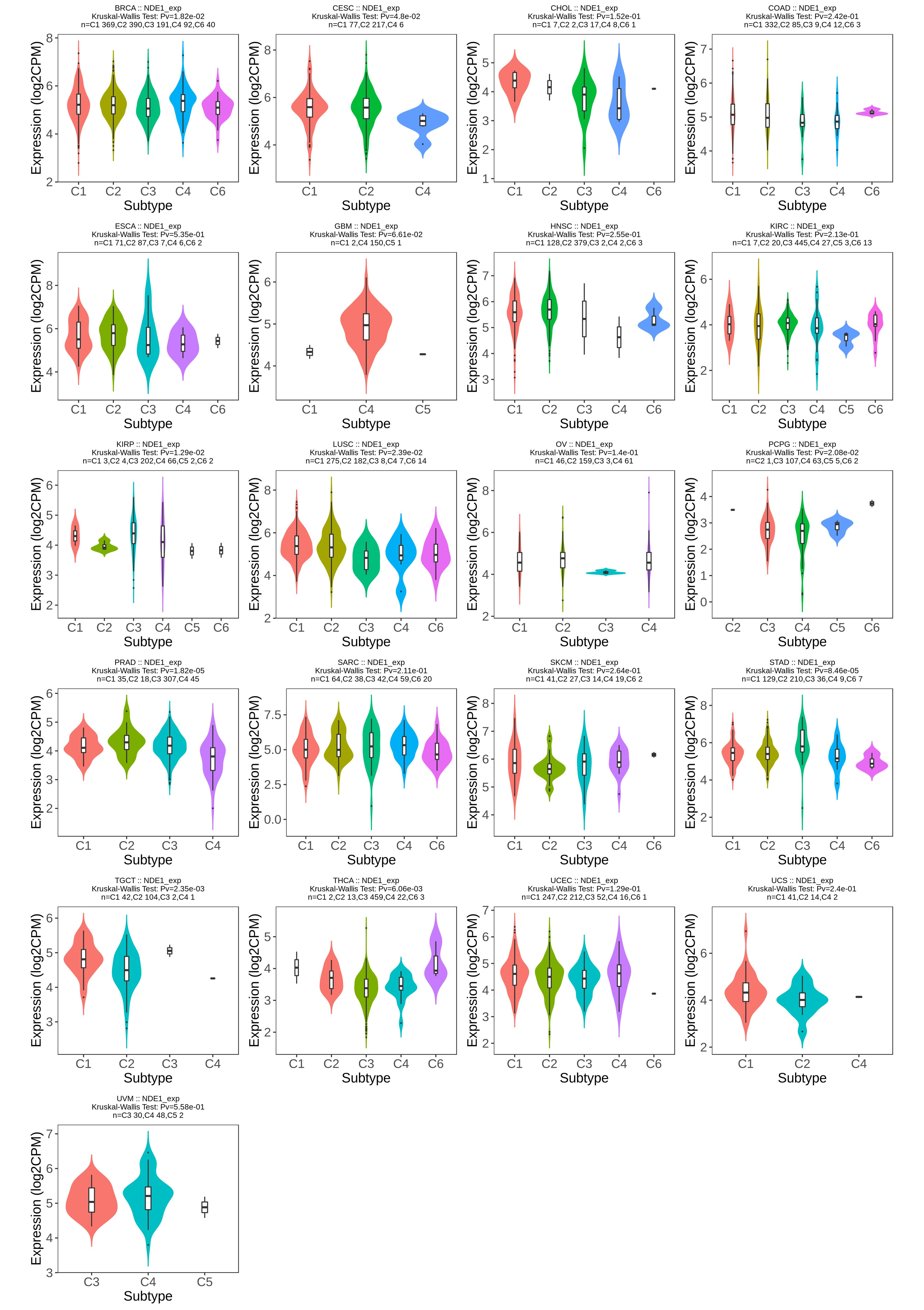

Supplement: Supplementary file 6 — Supplementary Figure S6. [file CAM4-13-e6931-s008.tif]

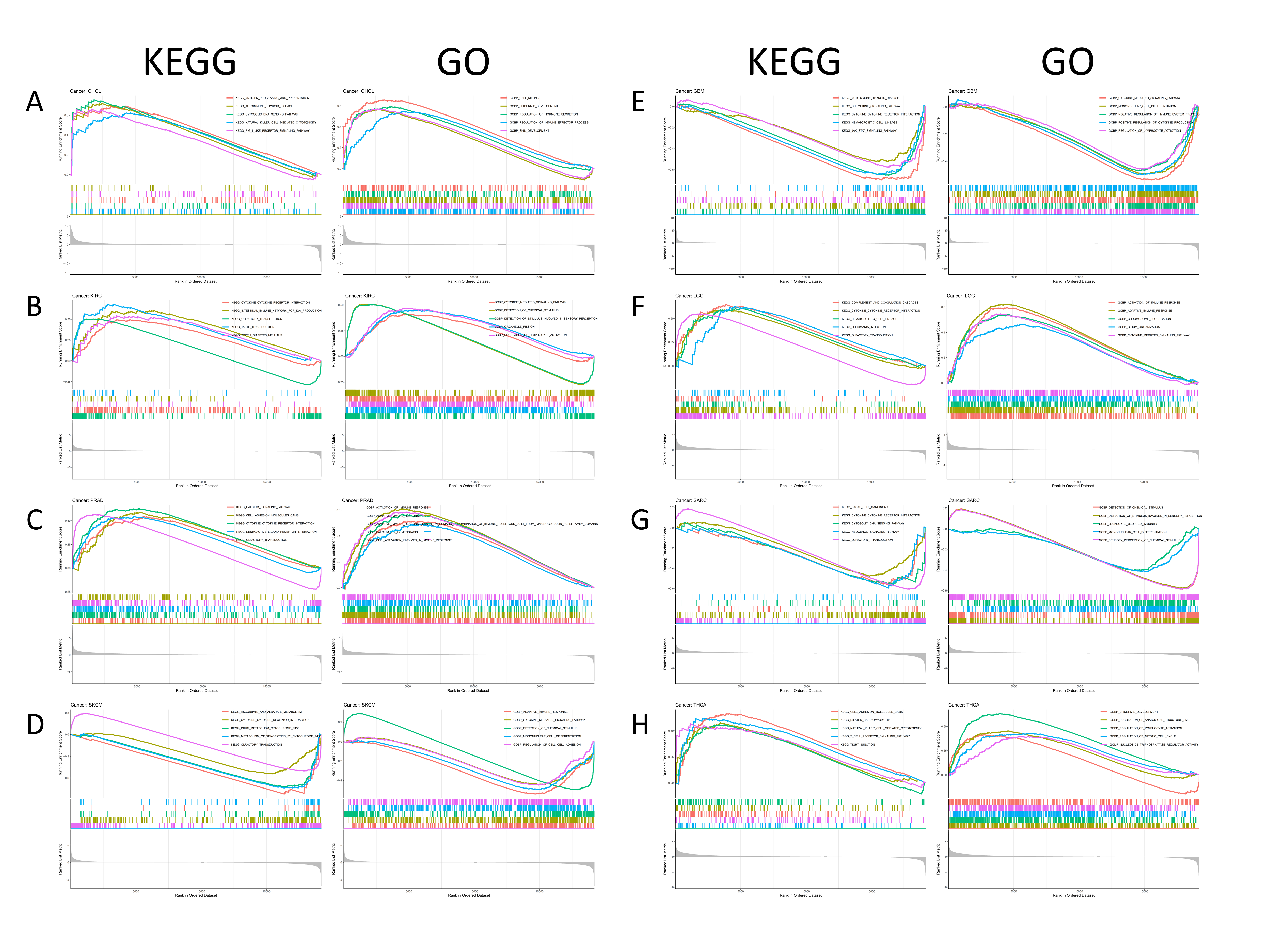

Supplement: Supplementary file 7 — Supplementary Figure S7. [file CAM4-13-e6931-s006.tif]

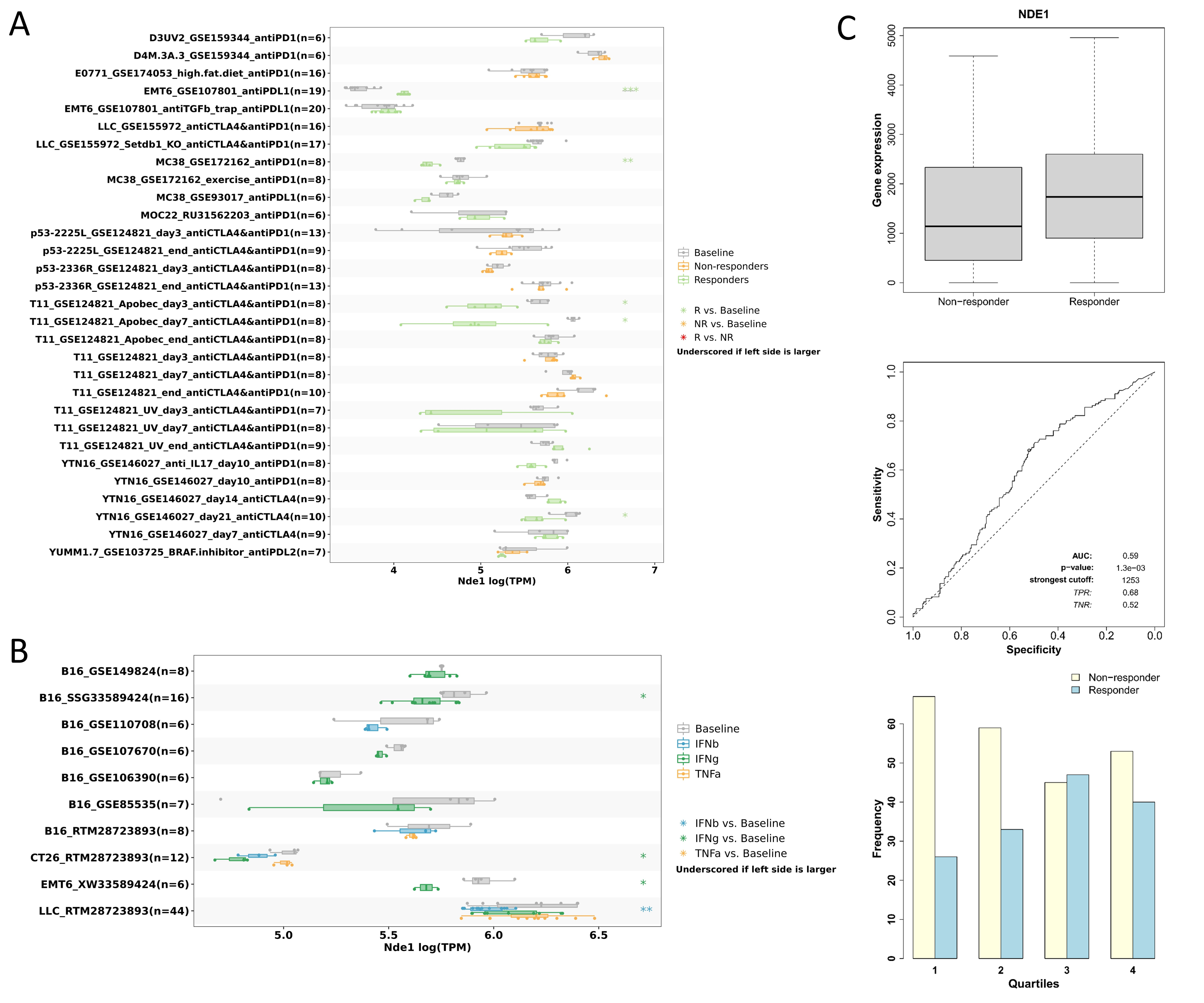

Supplement: Supplementary file 8 — Supplementary Figure S8. [file CAM4-13-e6931-s004.tif]
